# Rat PRDM9 shapes recombination landscapes, duration of meiosis, gametogenesis, and age of fertility

**DOI:** 10.1186/s12915-021-01017-0

**Published:** 2021-04-28

**Authors:** Ondrej Mihola, Vladimir Landa, Florencia Pratto, Kevin Brick, Tatyana Kobets, Fitore Kusari, Srdjan Gasic, Fatima Smagulova, Corinne Grey, Petr Flachs, Vaclav Gergelits, Karel Tresnak, Jan Silhavy, Petr Mlejnek, R. Daniel Camerini-Otero, Michal Pravenec, Galina V. Petukhova, Zdenek Trachtulec

**Affiliations:** 1 Laboratory of Germ Cell Development, Division BIOCEV, Institute of Molecular Genetics of the Czech Academy of Sciences, 14220 Prague, Czech Republic; 2 Laboratory of Genetics of Model Diseases, Institute of Physiology of the Czech Academy of Sciences, Prague, Czech Republic; 3 National Institute of Diabetes, Digestive, and Kidney Diseases, National Institutes of Health, Bethesda, MD 20892 USA; 4 Department of Biochemistry and Molecular Biology, Uniformed Services University of Health Sciences, Bethesda, MD 20814 USA; 5 Present address: Inserm U1085 IRSET, 35042 Rennes, France; 6 Institut de Génétique Humaine, CNRS UMR 9002, 34396 Montpellier, France; 7 Present address: Division BIOCEV, Laboratory of Epigenetics of the Cell Nucleus, Institute of Molecular Genetics of the Czech Academy of Sciences, 14220 Prague, Czech Republic; 8 Laboratory of Mouse Molecular Genetics, Division BIOCEV, Institute of Molecular Genetics of the Czech Academy of Sciences, 14220 Prague, Czech Republic

**Keywords:** *Rattus norvegicus*, Meiotic recombination, PRDM9, Fertility

## Abstract

**Background:**

Vertebrate meiotic recombination events are concentrated in regions (hotspots) that display open chromatin marks, such as trimethylation of lysines 4 and 36 of histone 3 (H3K4me3 and H3K36me3). Mouse and human PRDM9 proteins catalyze H3K4me3 and H3K36me3 and determine hotspot positions, whereas other vertebrates lacking PRDM9 recombine in regions with chromatin already opened for another function, such as gene promoters. While these other vertebrate species lacking PRDM9 remain fertile, inactivation of the mouse *Prdm9* gene, which shifts the hotspots to the functional regions (including promoters), typically causes gross fertility reduction; and the reasons for these species differences are not clear.

**Results:**

We introduced *Prdm9* deletions into the *Rattus norvegicus* genome and generated the first rat genome-wide maps of recombination-initiating double-strand break hotspots. Rat strains carrying the same wild-type *Prdm9* allele shared 88% hotspots but strains with different *Prdm9* alleles only 3%. After *Prdm9* deletion, rat hotspots relocated to functional regions, about 40% to positions corresponding to *Prdm9*-independent mouse hotspots, including promoters. Despite the hotspot relocation and decreased fertility, *Prdm9*-deficient rats of the SHR/OlaIpcv strain produced healthy offspring. The percentage of normal pachytene spermatocytes in SHR-*Prdm9* mutants was almost double than in the PWD male mouse oligospermic sterile mutants. We previously found a correlation between the crossover rate and sperm presence in mouse *Prdm9* mutants. The crossover rate of SHR is more similar to sperm-carrying mutant mice, but it did not fully explain the fertility of the SHR mutants. Besides mild meiotic arrests at rat tubular stages IV (mid-pachytene) and XIV (metaphase), we also detected postmeiotic apoptosis of round spermatids. We found delayed meiosis and age-dependent fertility in both sexes of the SHR mutants.

**Conclusions:**

We hypothesize that the relative increased fertility of rat versus mouse *Prdm9* mutants could be ascribed to extended duration of meiotic prophase I. While rat PRDM9 shapes meiotic recombination landscapes, it is unnecessary for recombination. We suggest that PRDM9 has additional roles in spermatogenesis and speciation—spermatid development and reproductive age—that may help to explain male-specific hybrid sterility.

**Supplementary Information:**

The online version contains supplementary material available at 10.1186/s12915-021-01017-0.

## Background

For sexual reproduction, animals generate haploid gametes via meiosis. To make these gametes unique, programmed double-strand DNA breaks (DSBs) are introduced and repaired by the recombination machinery using the homologous chromosomes as templates (reviewed in [1]). These meiotic DSBs are positioned by the histone-H3-lysine-4-trimethyl (H3K4me3) transferase PRDM9 in mice and men [2–5]. The mouse *Prdm9* gene is one of the factors responsible for intersubspecific mouse F1 hybrid sterility [6–8] due to changed DNA-binding specificity [9]. The changes in DNA binding by new PRDM9 alleles generate new recombination initiation sites and thus resolve the so-called meiotic hotspot paradox—the quick disappearance of the preferential DSB sites through their repair utilizing the homologous chromosome sequences, which do not contain the PRDM9-binding motifs [10]. Deficiency for *Prdm9* results in complete meiotic arrest in both sexes of the C57BL/6 J mouse (abbreviated as B6) [7, 11–13] and partial meiotic arrest in the male PWD/Ph mouse [14], accompanied by relocation of the DSBs into PRDM9-independent H3K4me3 sites (default sites) including gene promoters [14, 15]. These results lead to the hypothesis that PRDM9 protects functional genomic regions from the damage made by the recombination machinery [15]. However, most of the recombination initiation DSB sites are located near gene promoters in fungi and vertebrates that carry no *Prdm9* ortholog [16–18]. In addition, canids [19–22] and at least one human female [23] are fertile despite having a non-functional PRDM9-encoding gene. We therefore turned to another prominent rodent model, the rat, to evaluate the role(s) of *Prdm9* in gamete development. *Prdm9*-deficient rats were produced and phenotyped along with their littermate controls and the first genome-wide maps of the rat meiotic DSBs were generated. The DSBs in the *Prdm9*-deficient males of the SHR rat strain relocated into default H3K4me3 sites, many corresponding to DSB positions in *Prdm9*-deficient mice. However, unlike the B6 or PWD mice, some SHR rats of both sexes lacking the *Prdm9* function produced offspring. Nevertheless, we found that PRDM9 affects the length of meiosis and age of fertility in both sexes, as well as oogenesis and spermiogenesis. We conclude that the rat can execute meiotic recombination in PRDM9-independent sites, rendering it a suitable model for human meiotic studies. Moreover, the postmeiotic effect of PRDM9 on spermatogenesis widens the possibilities for the mechanism of PRDM9 action in hybrid sterility.

## Results

### Generation of the *Prdm9*-deficient rats

**Fig. 1:**
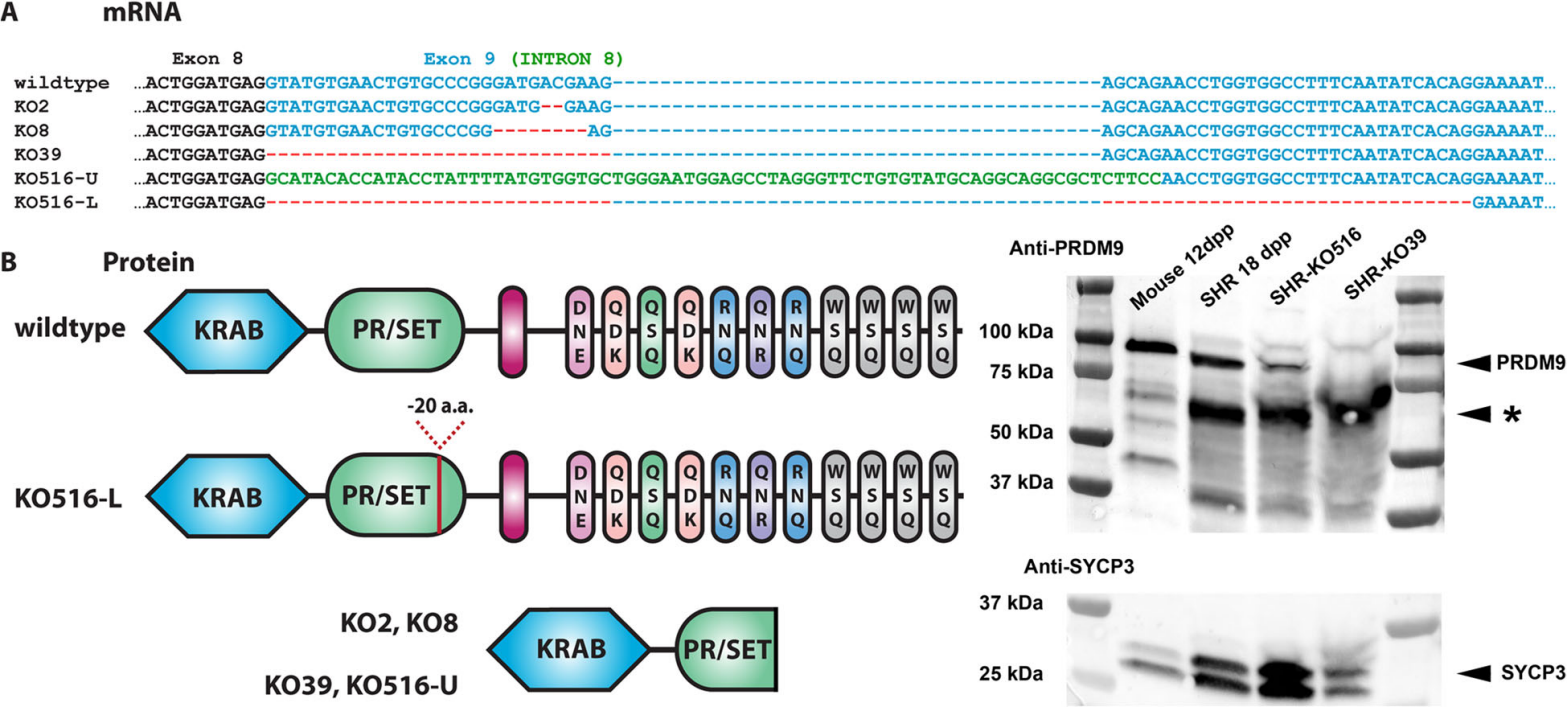
Four *Prdm9* deletions generated in SHR rats lead to mRNAs with open reading frame truncations and one also to alternative mRNA with deletion of 20 codons. **a** mRNAs arising from rats with genomic deletions of 2-, 8-, 39-, and 516-bp (abbreviated KO2, KO8, KO39, and KO516, respectively; the latter two also involve a part of Intron 8); blue dashes, gaps to optimize the alignment; red dashes, exonic deletions; -U, -L, two new mRNAs from the animal with the KO516 deletion. **b** Polypeptide products predicted from mRNAs in **a** and their detection. -20 a.a., deletion of 20 amino acid residues. The C-terminal boxes indicate zinc-fingers and the three letters their DNA-binding amino acids. See Additional file 1: Fig. S1 for details of the translations and amino acids essential for methyltransferase activity and Additional file 2: Table S1 for genomic and cDNA sequences. Right, PRDM9 was detected at the expected sizes of 97 kDa (mouse *Dom2* allele) and 96 kDa (rat SHR allele). Anti-SYCP3 antibody was utilized as a loading control. PRDM9 was present in the SHR-*Prdm9*^*KO516/KO516*^ mutants at the expected size of the KO516-L isoform (91 kDa), but its expression was lower than that of the wild-type in SHR; the KO516-U isoform (predicted 40 kDa) was undetectable. KO39 mutant product was not found (expected 44 kDa) in the SHR-*Prdm9*^*KO39/KO39*^ testes, thus no C-terminally truncated form of PRDM9 was seen in either mutant. Only a third of the amount of protein nuclear extract was loaded in mouse (10 μg) compared to rat (30 μg) lanes

Although some mammalian genomes carry multiple PRDM9-encoding genes, the rat genome was reported to contain only a single-copy *Prdm9* gene [24]. To decipher the necessity of *Prdm9* for rat fertility, we targeted this gene in the SHR/OlaIpcv strain (SHR; see Methods) in one of the exons encoding the PR/SET domain. This domain catalyzes both H3K4 [12] and H3K36 trimethylation in human and mouse [25, 26]. The mRNAs of a programmed heterodimeric nuclease were injected into 234 fertilized ova (Table 1). Four animals carrying deletions of two, eight, 39, and 516 base pairs in size (bp) were generated; the two largest ones also affected one exon-intron boundary. To assess the effects of these deletions on *Prdm9* mRNA, we performed reverse-transcribed (RT) PCR with primers from the exon sequences surrounding the deletions using testicular RNA. Sequencing of these RT-PCR products from testicular RNA revealed that the three shortest deletions resulted in the shifts of PRDM9 open reading frames, producing putative PR/SET domain truncations and exclusion of the PRDM9 zinc-finger DNA-binding domain (Fig. 1). The mutant with the 516-bp deletion produced two new transcripts, one with a frame shift and one with an internal deletion of 20 codons of the PR/SET catalytic domain that retained the PRDM9 zinc-finger-encoding exon in frame (Fig. 1, Additional file 1: Fig. S1). All deletions removed one or more amino acid residues important for the methyltransferase activity of PRDM9 ([27]; Additional file 1: Fig. S1). Rats genotyped homozygous for these mutations using genomic DNA were also homozygous when probed by RT-PCR using testicular mRNA, supporting whole genome sequence assembly data that suggested that *Prdm9* is present in a single copy in the rat [24, 28]. Semi-quantitative RT-PCR of testicular RNA revealed that males homozygous for the *Prdm9* deletions of 2-, 8-, and 39-bp display similar mRNA levels as littermate controls and confirmed expression of the two new mRNA isoforms from the mutant with the 516-bp deletion (Additional file 1: Fig. S2).

**Table 1:** Preparation of *Prdm9* mutant SHR animals by mRNA injections

Experiment	1	2	Total
mRNA concentration	2 ng/μl	5 ng/μl	Both
Injected zygotes	121	113	234
Transferred embryos 2c/1c*	57/18	62/21	119/39
Female recipients	6	7	13
Pregnant recipients	3	3	6
Live progeny born	15	8	23
Mutants	3	1	4

*2-cell stage/1-cell stage transferred into recipients after 20 h of in vitro culture post injection

To evaluate PRDM9 protein expression in wild-type and mutant testes, we performed Western blotting (Fig. 1b). PRDM9 was detected SHR testes at 18 days post partum (dpp), where most germ cells are pre/leptotene and zygotene spermatocytes (see below for details on 18-dpp testes). Neither PRDM9 protein nor its truncated form was found in 18-dpp SHR-*Prdm9*^*KO39/KO39*^ rat testes, suggesting that its frame-shifted mRNA is not translated or quickly degraded. PRDM9 was present in 18-dpp SHR-*Prdm9*^*KO516/KO516*^ rats at the size of 91 kDa predicted from the mRNA with the internal deletion of 20 codons of the catalytic PR/SET domain, but not at the size of 40 kDa expected from the alternatively spliced mRNA with the frame shift. However, the expression of the 91 kDa product was lower than the expression of the wild-type PRDM9.

### Inactivation of rat *Prdm9* is compatible with fertility in both sexes

**Fig. 2:**
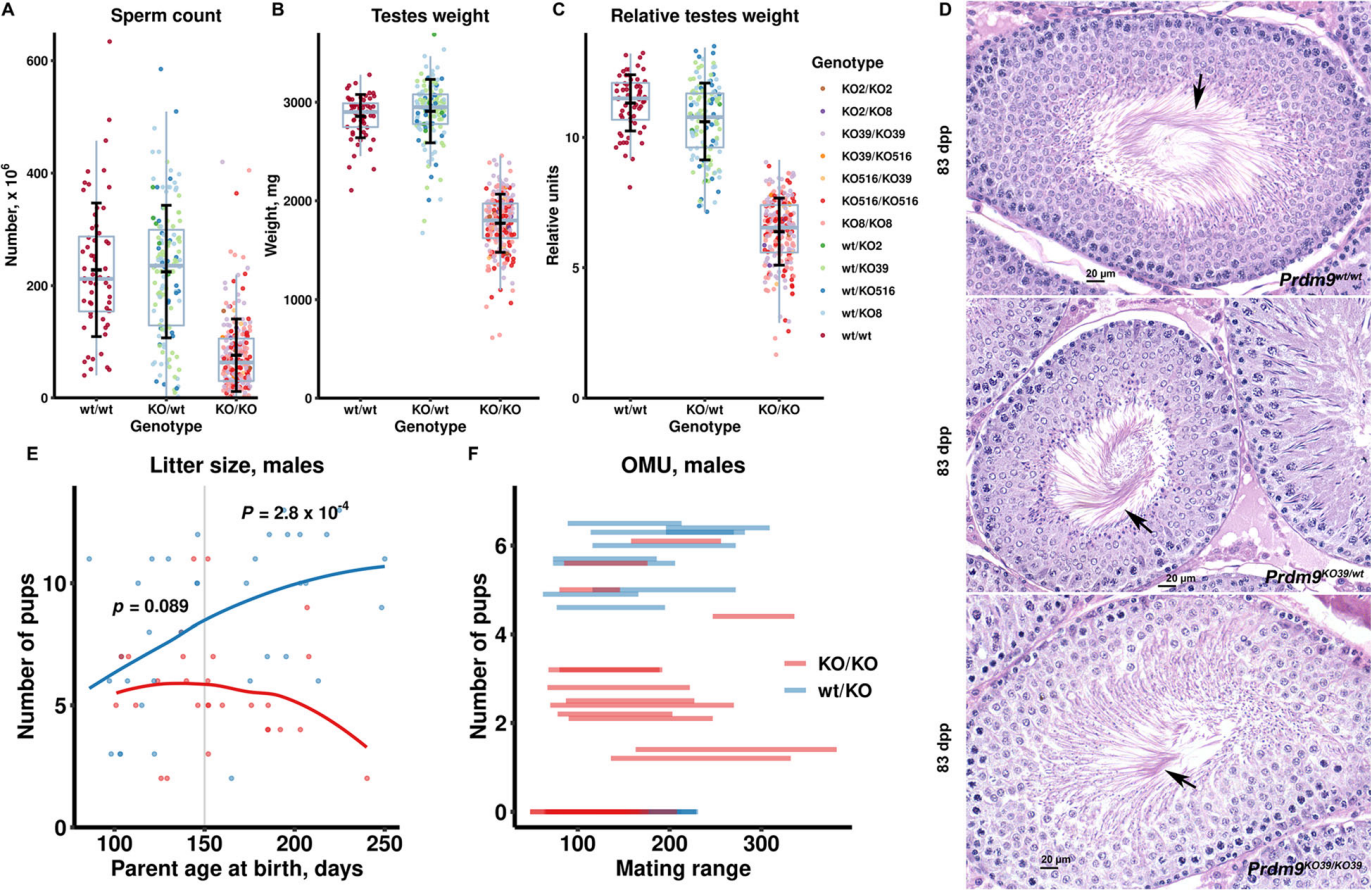
Decreased fertility parameters of SHR males carrying *Prdm9* deletions depend on age. Each dot depicts a single animal. **a–c** Boxplots of sperm count (**a**), testicular weight (**b**), and relative testicular weight (**c**, mg testicular weight per gram of body weight). **d**, Representative images of hematoxylin-eosin (HE) stained sections from rat testicles; note the presence of mature spermatids (arrows) in all three males. **e** Litter size of male parents of two indicated *Prdm9* genotypes for paternal age at birth. The statistical significance of the differences between genotypes was tested using LRM in the age groups of up to 150 and above 150 dpp. **f** Number of pups per month per female (OMU) for mating ranges (days) in SHR males of two indicated *Prdm9* genotypes. The data underlying all published plots are given in Additional file 3

We next tested the fertility of the rats carrying the *Prdm9* deletions. Twenty-one of 22 (95%) of *Prdm9*-deficient and all 26 tested heterozygous males sired offspring. Heterozygous *Prdm9*^*KO/wt*^ males that produced at least two litters (*n* = 26) sired 6.8 ± 1.6 (mean ± standard deviation) of offspring per female per month, 32% more compared to the *Prdm9*-deficient males with at least two litters (*n* = 15; 4.6 ± 2.1; *P* = 0.0009; Welsch’s *t* test; Fig. 2f). The litter size itself also decreased in *Prdm9*-deficient males (Fig. 2e). Adult (71 to 98 days old) *Prdm9*-deficient males had relative testicular weight and sperm count reduced by about 40% compared to both heterozygote and wild-type rats (*P* < 0.0001 each; linear regression model (LRM); Fig. 2a–c, Additional file 1: Fig. S3).

**Fig. 3:**
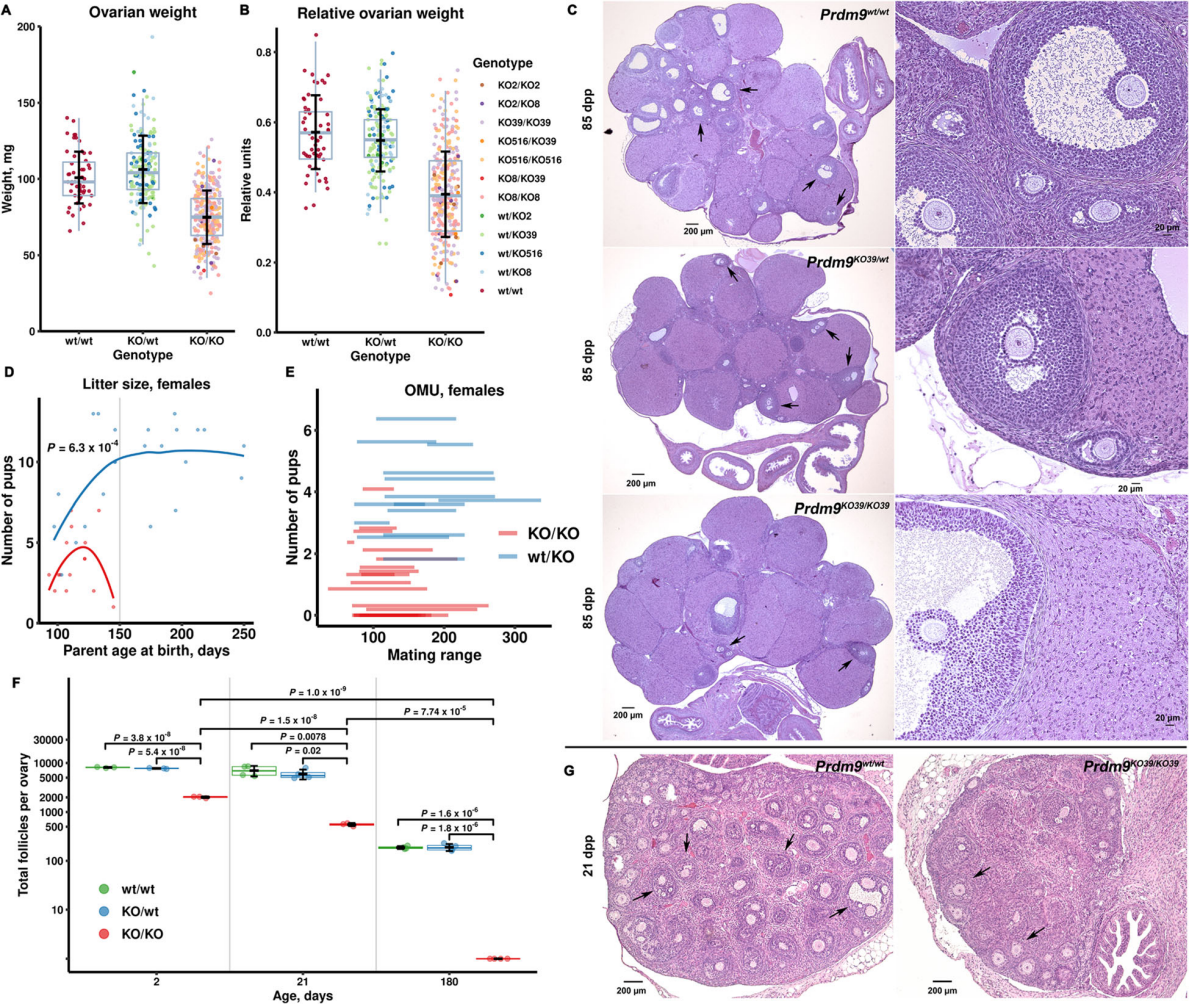
Decreased fertility and premature ovarian failure in SHR females carrying *Prdm9* deletions. **a**, **b** Boxplots of ovary weight (**a**, in mg) and of relative ovary weight (**b**, mg ovary weight per gram of body weight). **c** Examples of HE stained sections from three adult rat ovaries at two magnifications. Note that all females carry germ cell follicles (arrows). **d** Litter size of female parents of two indicated *Prdm9* genotypes with maternal age at birth. The difference between genotypes was tested using the LRM in the age group of up to 150 dpp. **e** Number of pups per month per female (OMU) and mating ranges (days) for two indicated *Prdm9* genotypes. The abscissa at OMU = 0 contains data for 14 *Prdm9*-deficient females, ten of which were mated after 150 dpp (for additional mean of 26 days). **f** Follicle quantification from ovarian sections of the indicated *Prdm9* genotypes at three points of postnatal development. Differences between three *Prdm9* genotypes and between animals of three ages within each genotype were analyzed using LRM with subsequent Tukey test and Bonferroni adjustment. **g** Representative images of HE-stained ovarian sections from 21-dpp females

*Prdm9*-deficient adult females (69 to 373 days old) had decreased ovary weight and relative ovary weight compared to controls (*P* < 0.0001, LRM accounting for age; Fig. 3a, b, Additional file 1: Fig. S3). Of 25 *Prdm9*-deficient females crossed for at least 2 months, 11 (44%, carrying all four deletion types) produced offspring, while all 27 tested *Prdm9*^*KO/wt*^ females yielded pups. Moreover, the litter size of the *Prdm9*-deficient *(Prdm9*^*KO/KO*^) female parents (5.4 ± 1.7) was smaller than that of *Prdm9*^*KO/wt*^ (8.3 ± 1.6; *P* = 0.0003; Welsch’s *t* test; Fig. 3d). The pups generated by both the male and female *Prdm9*^*KO/KO*^ rats were fertile and appeared normal. No offspring has been recovered when both mating partners were *Prdm9*-deficient (7 females, 2-month crosses). In summary, in contrast to B6 and PWD mice, both male and female SHR rats lacking PRDM9 function display only a partial reduction of fertility.

### Inactivation of rat *Prdm9* induces four partial arrests of spermatogenesis

To uncover the reasons for male fertility reduction, we performed immuno-, histo-, and cyto- chemistry of the germline. Within an adult rat testis, subsequent waves of spermatogenesis begin once every ~ 12 days (8–9 in the mouse) [29]; the entire rat spermatogenesis takes ~ 52 days. Premeiotic spermatogonia line the periphery of the seminiferous tubule, divide mitotically, and mature. The resulting primary spermatocytes proceed through meiotic prophase and two specialized divisions to yield spermatids, which differentiate into sperm until being released into the tubular lumen. Because a new spermatogenesis cycle begins before the previous cycles have ended, each tubule carries various germ cell types. As both the time between cycles and the duration of individual developmental stages are fixed, each rat tubule section can be classified into 14 types (12 in the mouse), referred to as stages I to XIV.

**Fig. 4:**
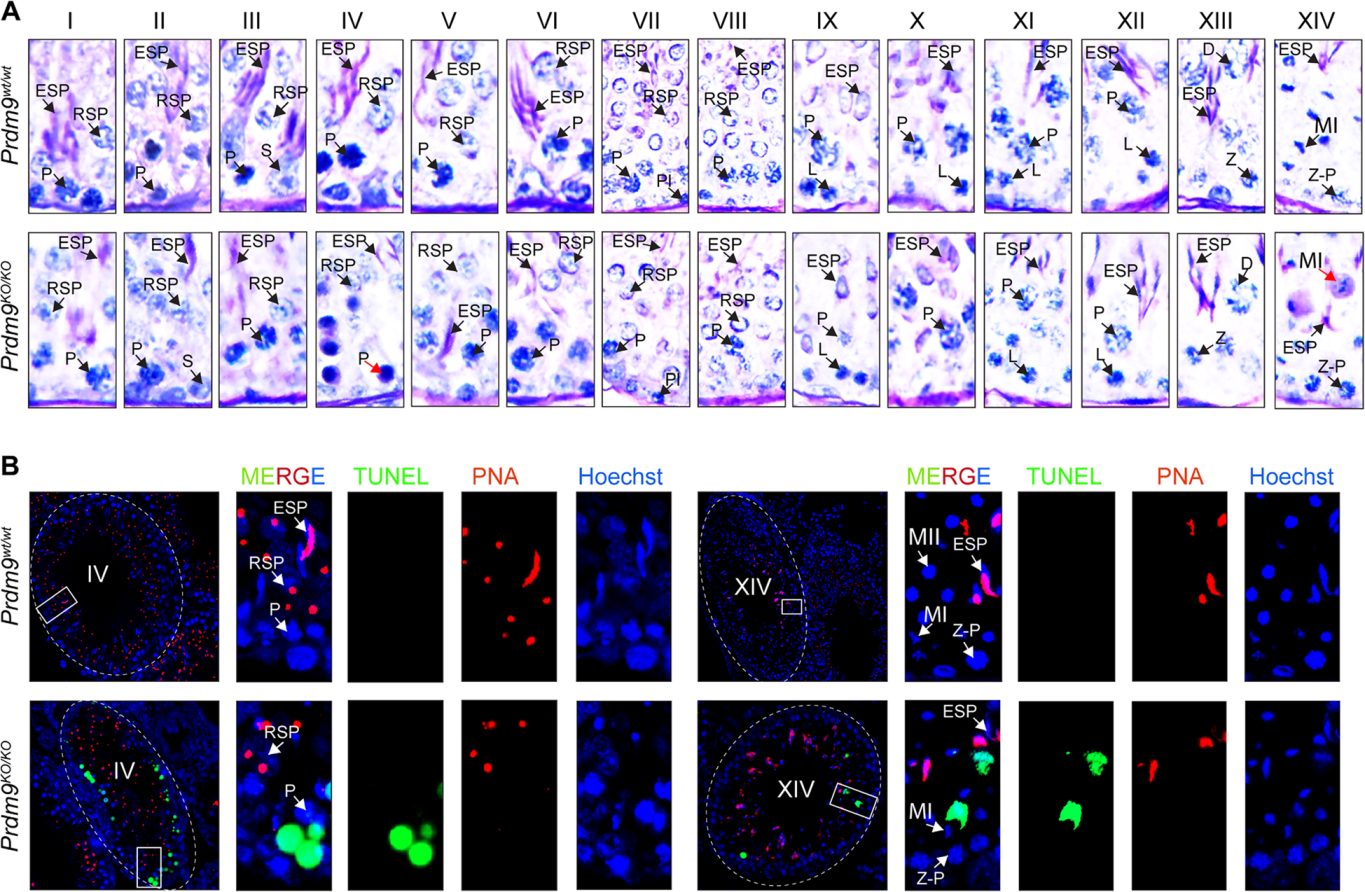
*Prdm9*-deficiency causes spermatogenic arrests in tubular stages IV and XIV of SHR rat. **a** Testicular histology using PAS-H staining. Illustrative examples of all 14 rat tubule stages. Degenerated cells in seminiferous tubules from adult (71 dpp) mutants stain intensively with PAS-H (blue-purple) at stages IV and XIV (red arrows). P, pachytene spermatocytes; RSP, round spermatids; ESP, elongated spermatids; S, Sertoli cells; Pl, preleptotene; L, leptotene; D, diplotene; Z-P, zygotene-pachytene spermatocytes; MI, metaphase I spermatocytes. Quantification of cells in all stages is presented in Fig. 5. **b** Increased apoptosis in mutant rat stage IV and XIV tubules confirmed by labeling apoptosis (TUNEL), acrosome (PNA), and DNA (Hoechst 33342)

**Fig. 5:**
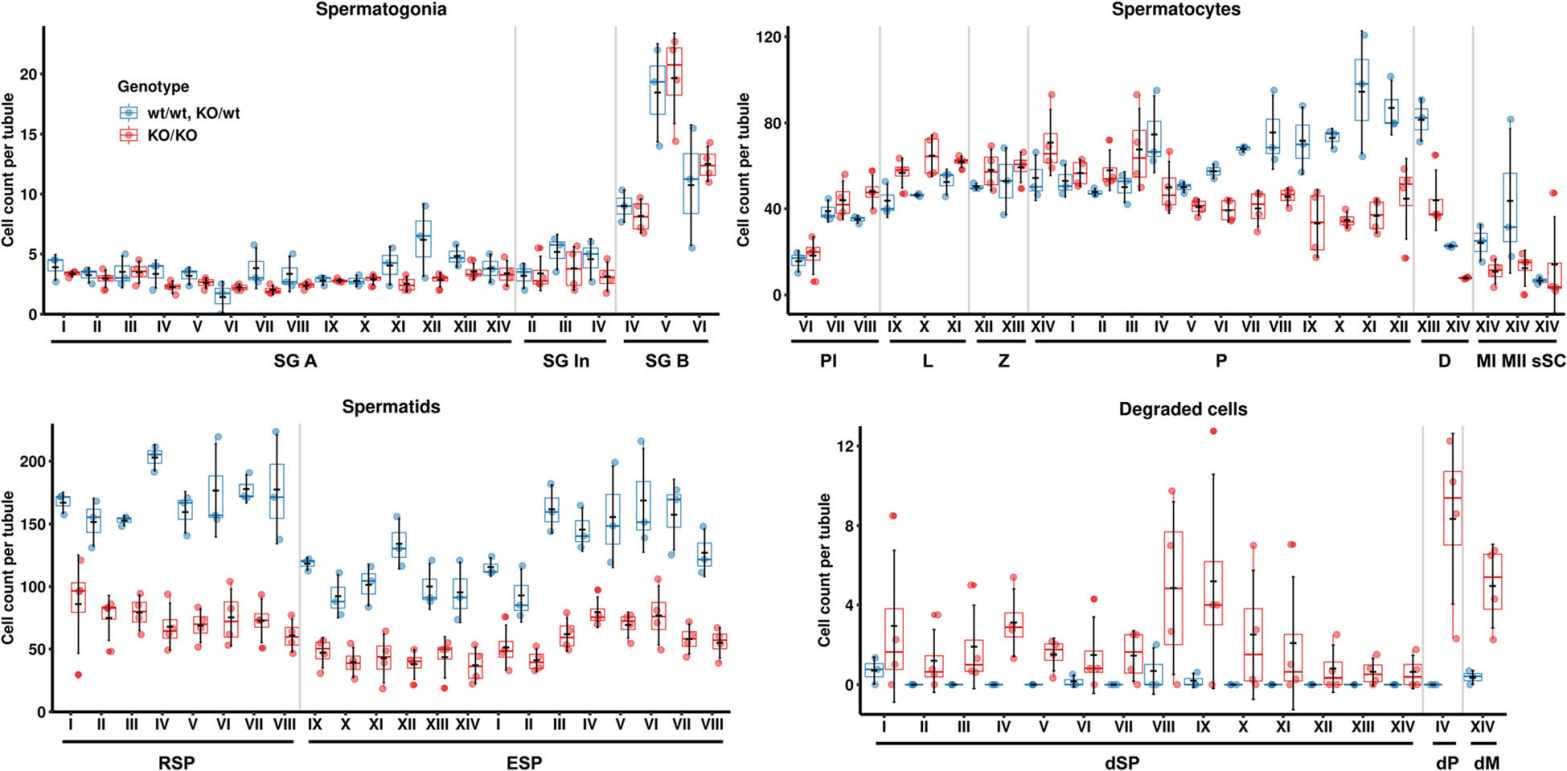
*Prdm9*-deficiency changes the quantities of spermatogenic cells in all 14 tubular stages of SHR rat. Cell counts of spermatogenic cell types in seminiferous tubules of stages I to XIV are given including the mean cell number ± standard deviation per tubule in each stage calculated from 3 to 5 tubules for each stage and animal (4 mutants and 3 controls) from sections stained with PAS-H. SG A, type A spermatogonia; SG In, intermediated spermatogonia; SG B, type B spermatogonia; see legend of Fig. 4 for abbreviations of primary spermatocytes and spermatids; MII, metaphase II spermatocytes; sSC, secondary spermatocytes; dM, degenerated MI/II and sSC; dP, degenerated pachytene; dSP, degenerated, halo, and joint spermatids

**Fig. 6:**
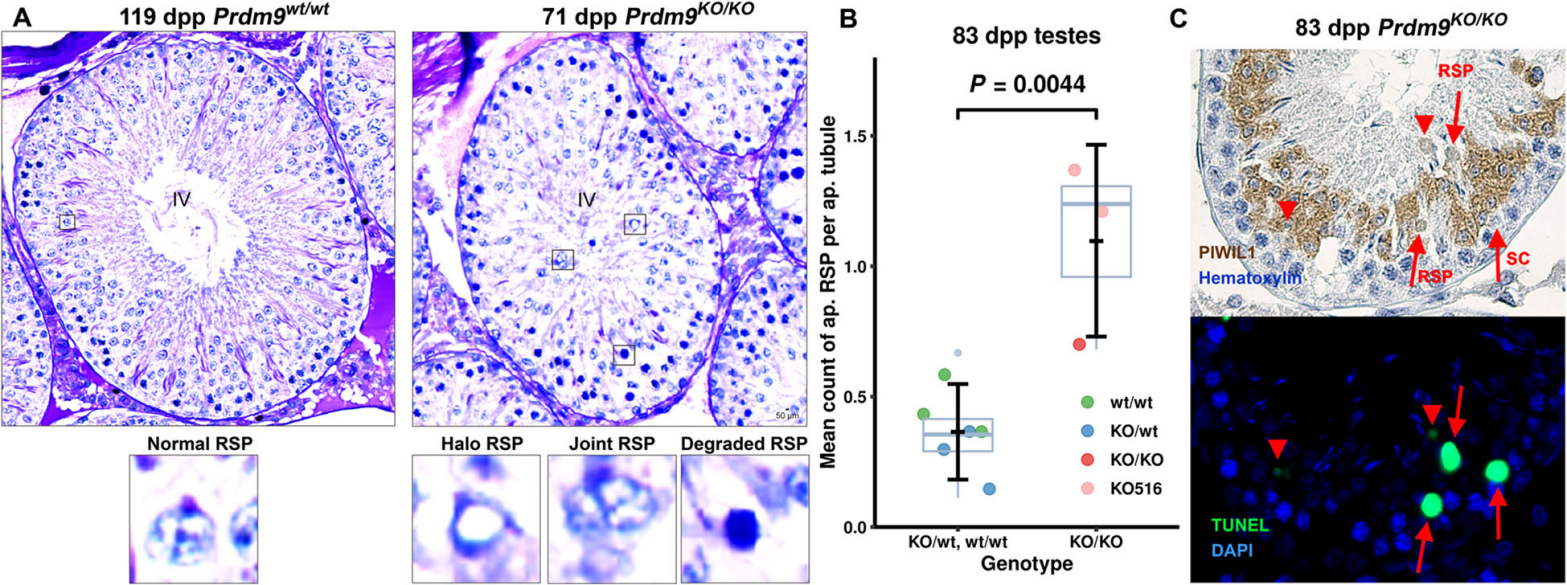
Rat PRDM9 affects postmeiotic development of round spermatids. **a** Examples of normal and defective spermatids from wild-type and *Prdm9*-deficient SHR males on PAS-H stained testicular sections. The means counted in mutant versus control in all 14 stages are in Fig. 5. **b, c** Increased apoptosis during postmeiotic development of *Prdm9*-deficient SHR testes. **b** Mean counts of apoptotic round spermatids per apoptotic tubule were scored from randomly chosen apoptotic tubules (18 to 32 per animal, total 221 tubules from 9 animals), plotted, and evaluated using LRM. The average counts of mutant and control samples were 1.1 ± 0.4 and 0.4 ± 0.2, respectively. **c** Round spermatids (RSP) were distinguished on neighboring TUNEL-DAPI and anti-PIWIL1-Hematoxylin-stained sections from spermatocytes (SC); representative images (the same tubule) are shown

To identify the specific stages of the seminiferous tubules in the mutant testes affected by *Prdm9* inactivation, we categorized and quantified cells in all 14 stages of rat tubules (over 60,000 cells) using sections from adult testes stained with periodic acid, Schiff, and hematoxylin (PAS-H, Fig. 4a and Fig. 5, statistics in Additional file 2: Table S2). Overall, all 14 tubule stages were readily identifiable and the counts of spermatogonia were similar in the mutant and control rat testes. However, the mutant tubules of all stages were more sparsely populated compared to controls (all 14 adjusted *P* values below 0.036; LRM), mostly due to lower counts of round and elongated spermatids (all 22 *P* < 0.037). These decreased numbers of spermatids could occur either solely due to partial meiotic arrest(s) or postmeiotic arrest(s) or both. To resolve these three possibilities, we compared abnormal cell counts. We found elevated counts of abnormal (halo [30], joint and degraded [31]) round spermatids in mutant stages IV (summary *P* = 0.027) and V (*P* = 0.028) compared to wild-type controls, which supports the contribution of the postmeiotic arrests (Fig. 6a). Moreover, we detected an increased number of defective mid-pachytene spermatocytes in stage IV (*P* = 0.022) and of degenerated metaphase spermatocytes in stage XIV (corresponding to mouse stage XII; *P* = 0.015) of *Prdm9*-deficient testes, indicative of two types of meiotic arrests. In agreement with this conclusion, the mutant stages V to XII contained less pachytene spermatocytes (all eight *P* values < 0.024) and mutant stage XIV both less late primary spermatocytes (*P* < 0.0001) and less metaphase II spermatocytes (*P* = 0.020) compared to the controls. In addition, stages VIII, X, and XI of mutant tubules contained elevated numbers of leptotene spermatocytes (all three *P* < 0.035), which may indicate prophase I delay. Altogether, the histopathology results suggested that the ablation of the rat *Prdm9* function caused partial arrests of meiosis at stages IV and XIV, a delayed meiotic prophase I, and incomplete spermiogenesis failures in stages IV and V.

To confirm these arrests of spermatogenesis, we inspected adult rat testicular sections for apoptosis using terminal deoxynucleotidyl transferase dUTP nick end labeling (TUNEL) and revealed that *Prdm9*^*KO/KO*^ had a 7-fold higher mean number of apoptotic cells per all tubules in comparison to *Prdm9*^*wt/wt*^ and *Prdm9*^*KO/wt*^ (*P* < 0.001 for both cases; LRM and Tukey’s test). To discern the germ cell types undergoing apoptosis, we combined these data with the results of anti-PIWIL1 immunohistochemistry (Fig. 6b, c). The PIWIL1 protein is expressed in pachytene spermatocytes and spermatids [32, 33]. Parallel analysis of 221 apoptotic tubules on neighboring TUNEL and anti-PIWIL1-hematoxylin-stained sections revealed that the majority of apoptotic cells (means of 82 to 88%, total 1059) were spermatocytes (*P* < 0.001 for both comparison of mutants to *Prdm9*^*wt/wt*^ and to *Prdm9*^*KO/wt*^). The mean number of apoptotic round spermatids per tubule in *Prdm9*^*KO/KO*^ also exceeded that in combined *Prdm9*^*KO/wt*^ and *Prdm9*^*wt/wt*^ (Fig. 6b, *P* = 0.0044), supporting the results of PAS-H staining. To further confirm the partial arrests of meiotic prophase I and metaphase, apoptotic cells were detected by the TUNEL assay on adult testicular sections co-labeled for acrosome (peanut agglutinin or PNA) and nucleus (Hoechst 33342) (Fig. 4b). Tubular stages IV and XIV in mutant testes carried more apoptotic cells (means of three testes 4.5 and 4.0%) than two wild-type controls (0.25 and 0.35%, respectively; both *P* < 0.007, generalized linear model or GLM). The close-to-complete mid-pachytene arrest present in B6-*Prdm9*^*KO/KO*^ mutants [14, 34] is mild in the SHR mutants. Thus, the decreased sperm count in the *Prdm9*-deficient rats can be explained by increased apoptosis during at least three tubular stages, IV (mid-pachytene spermatocytes and round spermatids), XIV (metaphase spermatocytes), and V (round spermatids).

### Rat PRDM9 is important for efficient spermiation

Mature rat spermatids (step 19 or S19) align at the luminal side of the seminiferous epithelium and are released into the tubular lumen during rat stage VIII in a process called spermiation [35]. To assess spermiation efficiency in *Prdm9*-deficient rats, we inspected the tubular stages IX and X from the rat testicular sections stained with PAS-H (Additional file 1: Fig. S4, Additional file 2: Table S3). Mature S19 wild-type SHR spermatids were rarely retained within the epithelium in stage IX (0.5 ± 0.4 per tubule), which contrasted with stage IX of *Prdm9*-deficient SHR (1.5 ± 0.2; *P* = 0.03, GLM). The S19 spermatid retention difference was also detected in wild-type versus mutant tubular stage X (0.2 ± 0.1 versus 1.8 ± 0.4; *P* = 0.01, GLM). These results suggested decreased spermiation efficiency. Thus increased apoptosis, abnormalities of round spermatids, and spermiation failure contribute to the reduced spermiogenic function of *Prdm9*-deficient rats. Altogether, our results show that PRDM9 is also required for normal rat haploid male gamete development and release.

### Inactivation of *Prdm9* leads to stage IV and XII arrests of PWD spermatocytes

To test the generality of two partial arrests of meiosis I at mid-pachytene and metaphase stages of *Prdm9*-deficient males, we used a *Prdm9*-deficient sterile PWD mouse male with gross reduction of sperm count [14]. We analyzed tubular stages IV and XII using PAS-H-stained testicular sections of adult *Prdm9*-deficient and control mouse PWD males (Additional file 1: Fig. S5, Additional file 2: Table S4). Comparing to wild-type PWD, we found increased numbers of degenerated pachytene spermatocytes in stage IV (4.9 ± 0.5 versus 0.0 ± 0.0; *P* < 0.001, GLM) and of metaphase cells in stage XII (3.2 ± 0.6 versus 0.7 ± 0.4; *P* < 0.001), suggesting that the two types of meiotic arrest reduce the numbers of *Prdm9*-deficient male germ cells.

Adult *Prdm9*-deficient PWD males display only 41% of wild-type pachytene spermatocytes with complete autosomal synapsis, while the rat SHR-*Prdm9*^*KO/KO*^ mutants display 72% of them, suggesting a weaker stage IV arrest in the rat. Consequently, we expected lower numbers of late primary spermatocytes in mouse tubular stage XII than in the corresponding rat stage XIV. Indeed, despite that the mean numbers of spermatogenic cells of all types in wild-type PWD mouse stage XII versus wild-type SHR rat stage XIV were similar, the mean counts of late primary spermatocytes were lower in the PWD mutant compared to the SHR mutant (4.1 ± 0.3 versus 7.7 ± 0.4), supporting the stronger mid-pachytene arrest in the PWD-*Prdm9*^*tm/tm*^ mutant. The sperm counts of the SHR and PWD mutants are about 33% and 1% of their corresponding wild-type counts. In agreement with these results, the number of elongated spermatids in stage IV from the SHR rat mutant were 17 to 37% and from the PWD mutant 0.3 to 3.9% of the SHR and PWD wild-type counts, respectively. In summary, both SHR rat and PWD mouse *Prdm9*-deficient males experience incomplete mid-pachytene and metaphase arrests.

### Most but not all rat *Prdm9*-deficient spermatocytes synapse homologous chromosomes

**Fig. 7:**
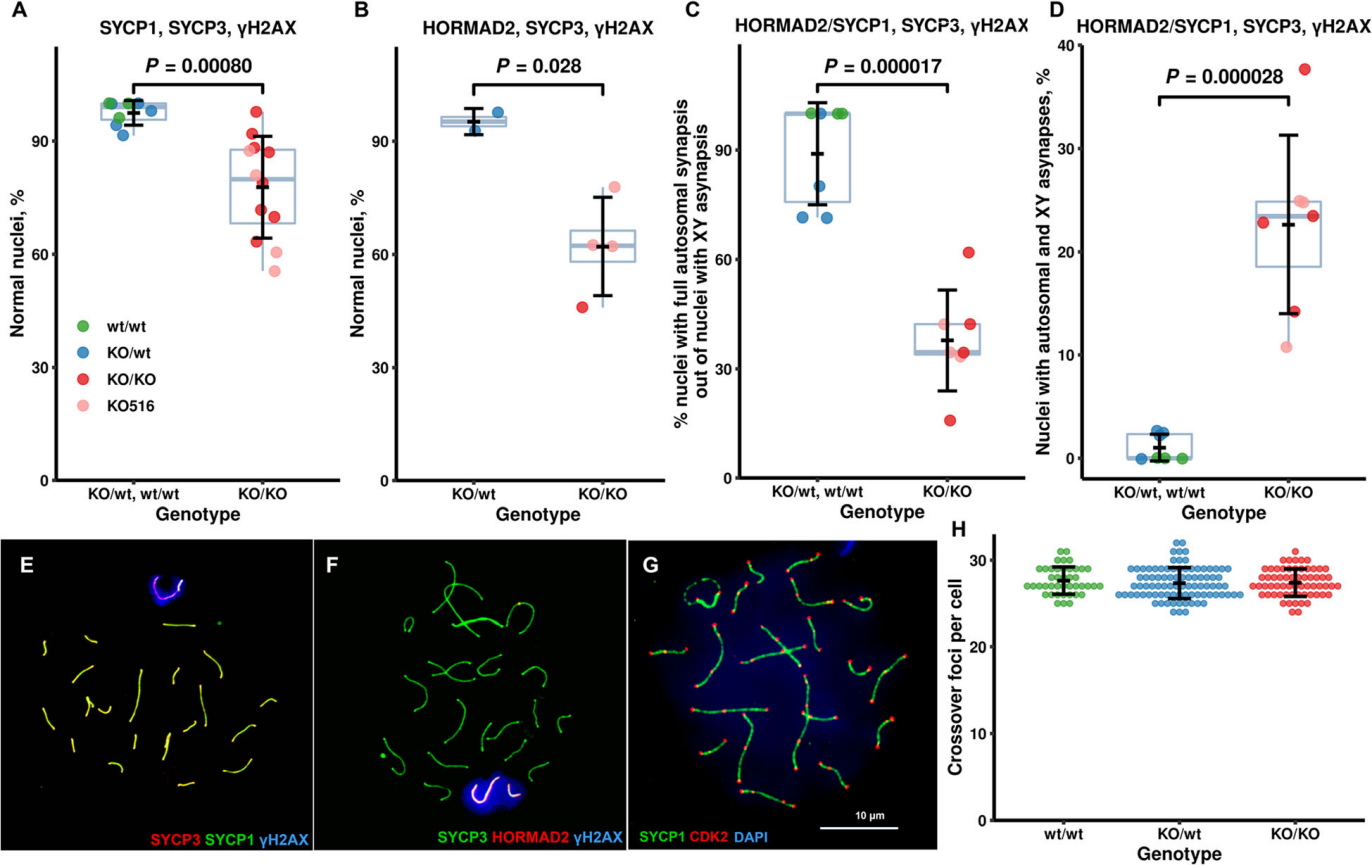
Decreased homologous synapsis but similar crossover rate in SHR males with *Prdm9* deletions compared to wild-type. **a**, **b** Percentage of normal pachytene spermatocytes (with all autosomes synapsed). Each dot represents a single animal (over 50 cells). Antibodies used for the staining of chromosomal spreads are given in the headings of each graph. Nuclear spreads used for the analysis of autosomal synapsis in both **a** and **b** were used for the analysis of XY synapsis in **c** and **d**. **c** Percentage of nuclei with full autosomal synapsis out of nuclei with XY asynapsis in *Prdm9*-deficient and control rat testes. **d** percentage of nuclei with both autosomal and XY asynapsis in *Prdm9*-deficient and control rat testes. **e**, **f**, Examples of normal pachytene cells. Immunocytochemistry with antibodies against: **e** Synaptonemal complex (SYCP1, SYCP3) and chromatin surrounding DSB sites (γH2AX); **f** SYCP3, γH2AX, and unsynapsed chromosomal axes (HORMAD2). See Additional file 1: Fig. S6 for the images of representative nuclei from *Prdm9*-deficient males. Differences between *Prdm9* genotypes were analyzed using LRM with subsequent Tukey test and Holm adjustment for multiple testing. **g** Representative image of chromosomal spread from a *Prdm9*-deficient rat immunostained for cyclin-dependent kinase 2 (CDK2) and synaptonemal complex (SYCP1), confirming that CDK2 localizes both to crossover nodules and telomeres as in the mouse. **h** Counts of autosomal crossover nodules per cell (*N* = 2 animals for both *Prdm9*^*KO/wt*^ and *Prdm9*^*KO/KO*^, *N* = 1 for wild-type)

As the B6-*Prdm9*^*KO/KO*^ mouse male displays complete and PWD-*Prdm9*^*KO/KO*^ partial pachytene arrest with persistent DSBs and incomplete synapsis [14, 34], we assayed adult rat pachytene nuclei by immunolabeling of the central element of the synaptonemal complex, the SYCP1 protein, and the DSB marker γH2AX (Fig. 7). The synaptonemal complex provides a scaffold for the alignment of homologous chromosomes and the central element keeps the homologs together through their entire length (homologous synapsis). Phosphorylated histone H2AX (γH2AX) is found in the vicinity of DSBs, but it also localizes to the X and Y chromosomes (sex body) at later meiotic stages (reviewed in [1]). Unlike *Prdm9*-deficient PWD males that carry 40% of normal (complete synapsis) pachytene spermatocytes [14], rat mutants displayed 78% (12 males, 729 nuclei) and wild-type SHR rats 96% normal pachytene spermatocytes (Fig. 7a). Four of 12 *Prdm9*-deficient males had a deletion of 20 codons in the PR/SET domain (516-bp deletion of total genomic DNA) and 72% normal pachynema while the other eight carried only truncating frameshift mutations (80%). There were no significant differences in the percentage of normal pachytene cells with fully synapsed autosomes between these four versus eight *Prdm9*-deficient rats (*P* = 0.09, GLM). Consistently, the testicular weights and sperm counts of these two *Prdm9* deletion types were also similar (Fig. 2). To confirm the role of rat PRDM9 in synapsis, chromosome spreads from adult testes were also labeled using antibodies against the HORMAD2 protein that decorates asynapsed homologous chromosomes during pachynema ([36]; Fig. 7b). Ninety-five percent of pachytene spermatocytes from *Prdm9*^*KO/wt*^ but only 62% from SHR-*Prdm9*^*KO/KO*^ animals were normal (*P* < 0.0001, GLM). Thus, rat SHR males require PRDM9 for efficient synapsis, but to a lesser degree than mouse PWD males and unlike B6 males, where PRDM9 is nearly essential for synapsis [14].

### Rat PRDM9 is important for efficient synapsis of sex chromosomes

To check whether PRDM9 also supports synapsis of rat sex chromosomes, we analyzed spread nuclei of pachytene spermatocytes from adult testes using immunolabeling (Fig. 7c, d). Seven mutant testes carried 192 of 549 (35%) and seven controls (3 wild-type and 4 heterozygous *Prdm9* males) 42 of 803 (5%) cells with XY asynapsis. Eighty-six percent (36 of 42) of control cells but only 35% (68 of 192; Fig. 7c) mutant cells displayed complete autosomal synapsis, thus probably being late pachynema. The remaining 124 *Prdm9*-deficient spermatocytes (23% of 549, 65% of 192) with both sex and autosomal asynapses were rare in the controls (0.7% of 803, 14% of 42; Fig. 7d) and represented aberrant cells. These results suggest that rat *Prdm9* is required for efficient XY synapsis.

### Rat PRDM9 supports repair of DSBs and does not affect the crossover rate

Mouse PRDM9 is important for efficient DSB repair [12, 34]. To assess the effect of PRDM9 on repair of meiotic DSBs in the rat, we analyzed spread testicular nuclei from *Prdm9*-deficient adult spermatocytes. Immunostaining for synaptonemal complex, centromeres, and early stages of DSB repair (RAD51/DMC1) revealed a few RAD51/DMC1 foci on autosomes and multiple foci on sex chromosomes in normal early pachytene spermatocytes (Additional file 1: Fig. S6m-p). All RAD51/DMC1 signal disappeared in normal late pachynema (Fig. S6q). However, abnormal cells displayed many strong RAD51/DMC1-stained foci on asynapsed chromosomes (Fig. S6r), suggesting that PRDM9 is important for efficient repair of programmed DSBs to crossovers.

Because the sperm presence in the mouse lacking *Prdm9* function correlates with crossover rate [14], we analyzed the number of meiotic crossovers in rats with different *Prdm9* copy numbers, including SHR-*Prdm9*^*wt/wt*^ and SHR-*Prdm9*^*KO/KO*^ males. Spermatocytes were immunostained with antibodies to the CDK2 protein that localizes to crossover sites in mouse meiotic prophase ([37]; Fig. 7g). All *Prdm9*-deficient spermatocytes with SYCP1 and CDK2 signals found (63 from two males) were fully synapsed. Both *Prdm9*-deficient males tested carried on average 27.4 autosomal crossovers per cell, the same as three control males (27.4 ± 0.2; Fig. 7h), indicating no effect of *Prdm9* on the mean crossover rate of rat males.

### The reduced fertility parameters of *Prdm9*-deficient rats change with age

**Fig. 8:**
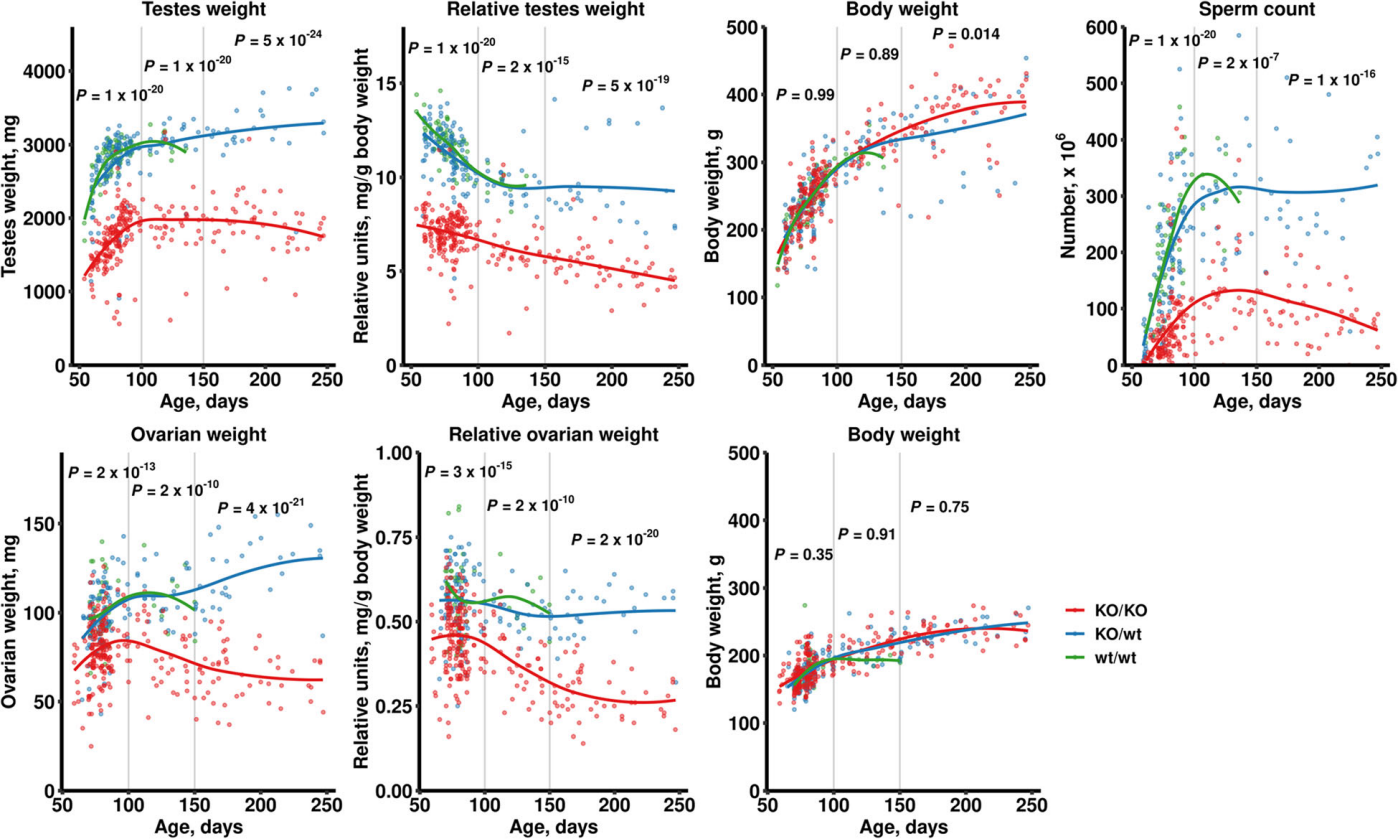
Fertility parameters of *Prdm9*-deficient animals change with age. Differences between three *Prdm9* genotypes were analyzed using LRM with subsequent Tukey tests and Holm adjustment in three age groups: 50–100, 100–150, and 150–250 dpp. *P* values illustrate differences between SHR-*Prdm9*^*KO/wt*^ and SHR-*Prdm9*^*KO/KO*^ rats. The comparison of wild-type and SHR-*Prdm9*^*KO/wt*^ rats revealed no differences in fertility parameters and had highly consistent correlation curves. The correlation between age and body weight was used as a negative control

*Prdm9* affects delayed fertility of intersubspecific mouse hybrid males [6]. To uncover the age-dependent effects of *Prdm9* on rat fertility, we analyzed various fertility parameters at multiple time points up to 250 dpp. The *Prdm9*-deficient animals displayed lower fertility parameters at all ages. Except for body weight used as negative control, all fertility parameters tested started to decline in SHR-*Prdm9*^*KO/KO*^ but not in control animals after about 100–150 dpp (Fig. 8).

To assess male fertility more directly, we crossed the rat males and analyzed the offspring production (Fig. 2e, f). In agreement with their testicular weight and sperm counts, the *Prdm9*-deficient males sired less pups after 150 dpp, thus validating the effect of PRDM9 on age-dependent fertility.

### Inactivation of rat *Prdm9* leads to premature ovarian failure

PWD and B6 *Prdm9*-deficient mouse females are sterile, as they form very few follicles and lose all oocytes before adulthood [12, 14]. To explain the decreased ovarian weight of the *Prdm9*-deficient rats, we checked the effect of *Prdm9* on follicular development and age of fertility. Folliculogenesis starts with formation of rat primordial follicles on 2 dpp. Our analysis revealed 25% primordial follicles in SHR-*Prdm9*^*KO/KO*^ compared to control females at 2 dpp (Fig. 3f), indicating that the majority of oocytes are lost before the onset of follicular development. Primary, secondary, and small antral follicles can be observed beside primordial follicles in juvenile rats before the onset of estrus on 21 dpp. The mean total number of follicles in the mutant was only 7% of controls at 21 dpp and almost no primordial follicles were detected. This decreased size of the oocyte pool likely contributes to a decreased littersize. *Prdm9*-deficient rats contained no follicles at 180 dpp, which contrasted with the controls (Fig. 3f, g). The paucity of oocytes explains the lack of offspring in mutant females after 150 dpp (Fig. 3d), despite being mated at this age (Fig. 3e), and it is also reflected by the decreasing ovarian weight (see above). These phenotypes of the SHR-*Prdm9*^*KO/KO*^ females resemble the human Premature Ovarian Failure syndrome, where the oocyte pool is exhausted early in age leading to sterility [38]. Altogether, these results suggest the importance of rat *Prdm9* for oocyte survival and follicle development.

### Rat PRDM9 affects the duration of meiotic prophase I in both sexes

**Fig. 9:**
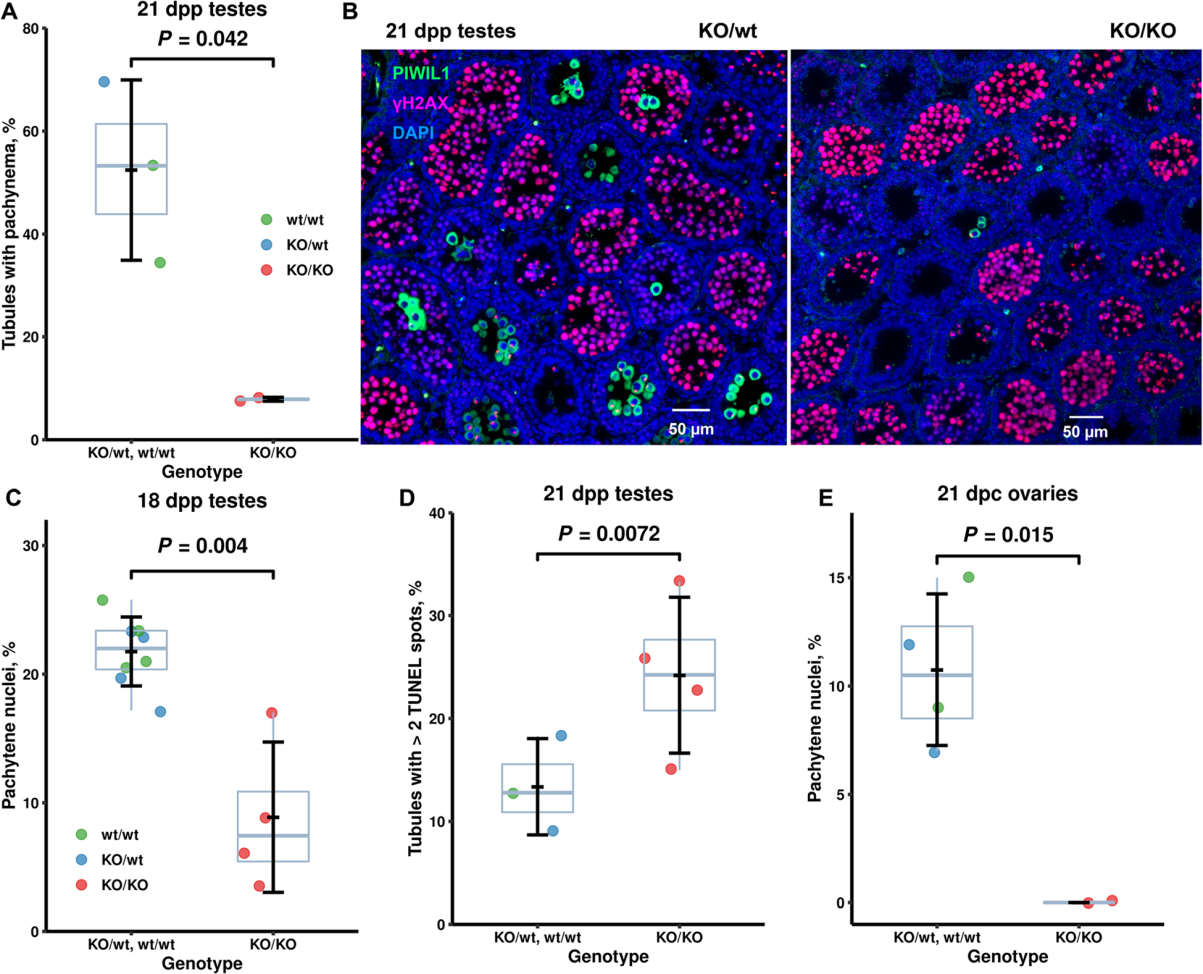
Delayed meiosis in male and female SHR rats lacking *Prdm9* function. **a-d** Delayed prophase I in *Prdm9*-deficient male rats compared to controls. **a**, **b** Immunohistochemistry of 21-dpp testes (154 to 368 tubules scored from each animal). **c** Immunocytochemistry of 18-dpp testicular spreads (over 400 spermatocytes for each genotype). **d** Delayed meiosis is associated with apoptosis. Immunohistochemistry of prepubertal testes using TUNEL and DAPI. **e** Delayed onset of pachynema in *Prdm9*-deficient female rats. Spread 21-dpc ovarian nuclei were staged using immunocytochemistry (over 250 oocytes scored for each of the three *Prdm9* genotypes; LRM). The data underlying all published plots are in Additional file 3

Partial meiotic arrest in semifertile mouse hybrids, which is alleviated by manipulating *Prdm9* dosage, is accompanied by meiotic delay [6] and SHR-*Prdm9*^*KO/KO*^ adult males displayed increased leptotene counts compared to controls in several tubular stages. To validate the requirement of *Prdm9* for normal meiotic progression kinetics in SHR males, we analyzed the first wave of rat spermatogenesis by immunohisto- and cytochemistry. We inspected sections of prepubertal PRDM9-deficient and control (wild-type and *Prdm9*^*KO/wt*^) SHR testes immunolabeled for chromatin surrounding DNA breaks under repair (γH2AX) and cytoplasmic piwiRNA particles (PIWIL1). Fifty-two percent of testicular tubules in 21-dpp controls (3 males, 154 to 368 tubules scored) but only 8% tubules of their mutant littermates (565 tubules from two animals) were classified as containing cells in pachynema (Fig. 9a, b; *P* = 0.042).

To confirm the meiotic delay found at the tissue level also on cells, we immunostained spreads of testicular nuclei from 18-dpp males for synaptonemal complex markers. Pachytene spermatocytes formed about 8% of all spermatocytes in the mutant testes compared to 21% in controls (Fig. 9c). To evaluate the association of the mutant rat meiotic prophase I delay with partial meiotic arrest, we inspected testicular sections from 21-dpp SHR males for apoptosis using TUNEL staining. There was a higher percentage of tubules containing multiple apoptotic cells in the mutant compared to control testes (means of 24 versus 13%), suggesting a weak arrest of prophase I (Fig. 9d). This conclusion is supported by the analysis of PAS-H staining and apoptosis in tubular stage IV of adult rat mutant testes (see above).

Both male and female *Prdm9*-deficient SHR animals displayed age-dependent fertility. To evaluate the importance of PRDM9 for the timely meiotic progression in SHR females, we staged spread nuclei from embryonic ovaries, where the single wave of female meiotic prophase I takes place (Fig. 9e). We used markers of synaptonemal complex (SYCP3 and SYCP1) and chromatin surrounding DNA breaks under repair (γH2AX). Seven to 15% oocytes in four pairs of control ovaries at 21 days post coitum (dpc) were pachytene. However, two pairs of littermate mutant ovaries contained no pachytene nuclei (Fig. 9e; *P* = 0.015, LRM), validating the prophase I delay of female rat meiosis in the absence of PRDM9.

Our results show that PRDM9 deficiency delays the onset of pachytene stage and slows down the progression of meiotic prophase I in SHR rats of both sexes.

### Rat PRDM9 trimethylates H3K4 and guides most meiotic DSBs

**Fig. 10:**
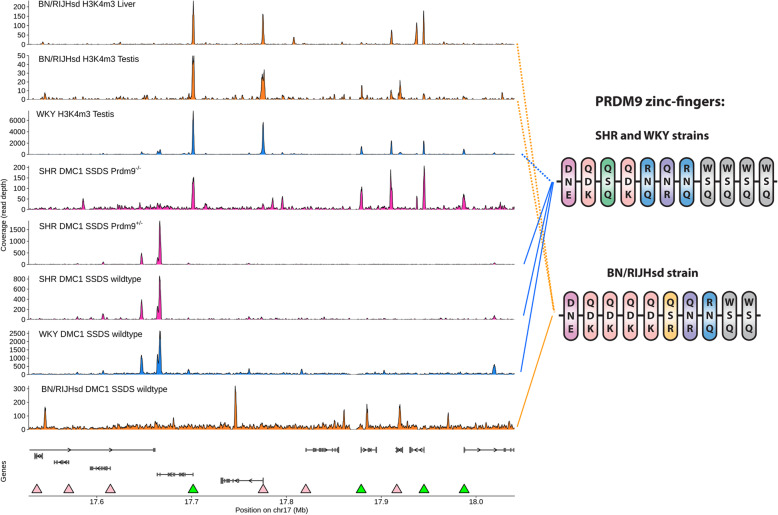
The positions of the rat meiotic DSB hotspots are determined by PRDM9. Left, a region of rat Chromosome 17 (rn5 assembly) exemplifying that the DSB hotspots (shown as peaks) in wild-type SHR males (SHR *Prdm9*^+/+^ SSDS) are shared with the strain with the same *Prdm9* allele (WKY *Prdm9*^+/+^ SSDS), but not with the strain with different *Prdm9* allele (BN/RIJHsd *Prdm9*^+/+^ SSDS) or inactivated *Prdm9* allele (SHR *Prdm9*^*−/−*^ SSDS). DSB hotspots in *Prdm9*-deficient male co-localize with testicular (T) and less with liver (L) H3K4me3 marks. The H3K4me3 profiles from the BN a WKY strains are different and reflect hotspot sites unique to each of these strains, thus validating that rat PRDM9 displays H3-methyltransferase activity. Some promoters correspond to *Prdm9*-independent hotspots (green arrowheads), but other promoters (pink arrowheads) are not targeted. The row “Genes” shows Ensembl gene models. Raw ChIP-seq coverage is shown in 150-bp windows; each panel is scaled to the maximum value. Right, DNA-binding zinc-finger arrays of PRDM9 from two rat strains showing polymorphic amino acid residues in contact with DNA

In order to assess the function of rat PRDM9 in DSB positioning, genome-wide distributions of DSBs were analyzed in two related rat strains carrying the same *Prdm9* allele, WKY and SHR, and in the BN/RIJHsd strain that harbors a distinct *Prdm9* allele (Fig. 10, right) by single-stranded DMC1-bound DNA sequencing (SSDS). The identified DSB hotspots overlapped by 88% between the SHR and WKY strains, but only by 3% between the BN and WKY strains (Fig. 10, Additional file 1: Fig. S7). DSB hotspots were stronger on chromosome X than on autosomes (Additional file 1: Fig. S8), as expected [15]. The strain overlap data suggest that PRDM9 controls most sites of rat recombination initiation. To address the epigenetic function of rat PRDM9, we generated H3K4me3 profiles from the BN and WKY testes. The profiles differed at variable hotspot sites (Fig. 10), thus validating that rat PRDM9 displays in vivo H3K4-methyltransferase activity. Relocation of meiotic DSBs to H3K4me3 at promoters and other functional genomic elements in the *Prdm9* knockout B6 mice was suggested as a possible cause of infertility. The alleviation of the infertile phenotype in the *Prdm9*-deficient rats could therefore be caused by the existence of another factor directing the recombination machineries away from these sites. The existence of this factor was hypothesized based on the analysis of the sites of PRDM9-independent human crossovers [23]. To check this possibility, genome-wide distribution of meiotic DSBs was compared between an adult SHR male carrying the homozygous KO39 truncating deletion (Fig. 1) and its wild-type littermate. The distribution of DSB hotspots in rats lacking PRDM9 was similar to that in mice: a large percentage of hotspots coincided with the default H3K4me3 marks, many of which were at gene promoters (Fig. 10 and see below). The relocation of DSB hotspots in the *Prdm9*-deficient rat to the default H3K4me3 sites (98.6% of 21,586 SHR DSB hotspots) and the similarity of the phenotypes of the four rat *Prdm9* deletions support the view that the truncating mutations result in null alleles. Therefore, it is unlikely that the rat has other factors besides PRDM9 to avoid recombination at default H3K4me3 sites.

### *Prdm9*-independent hotspots tend to localize to orthologous regions

**Fig. 11:**
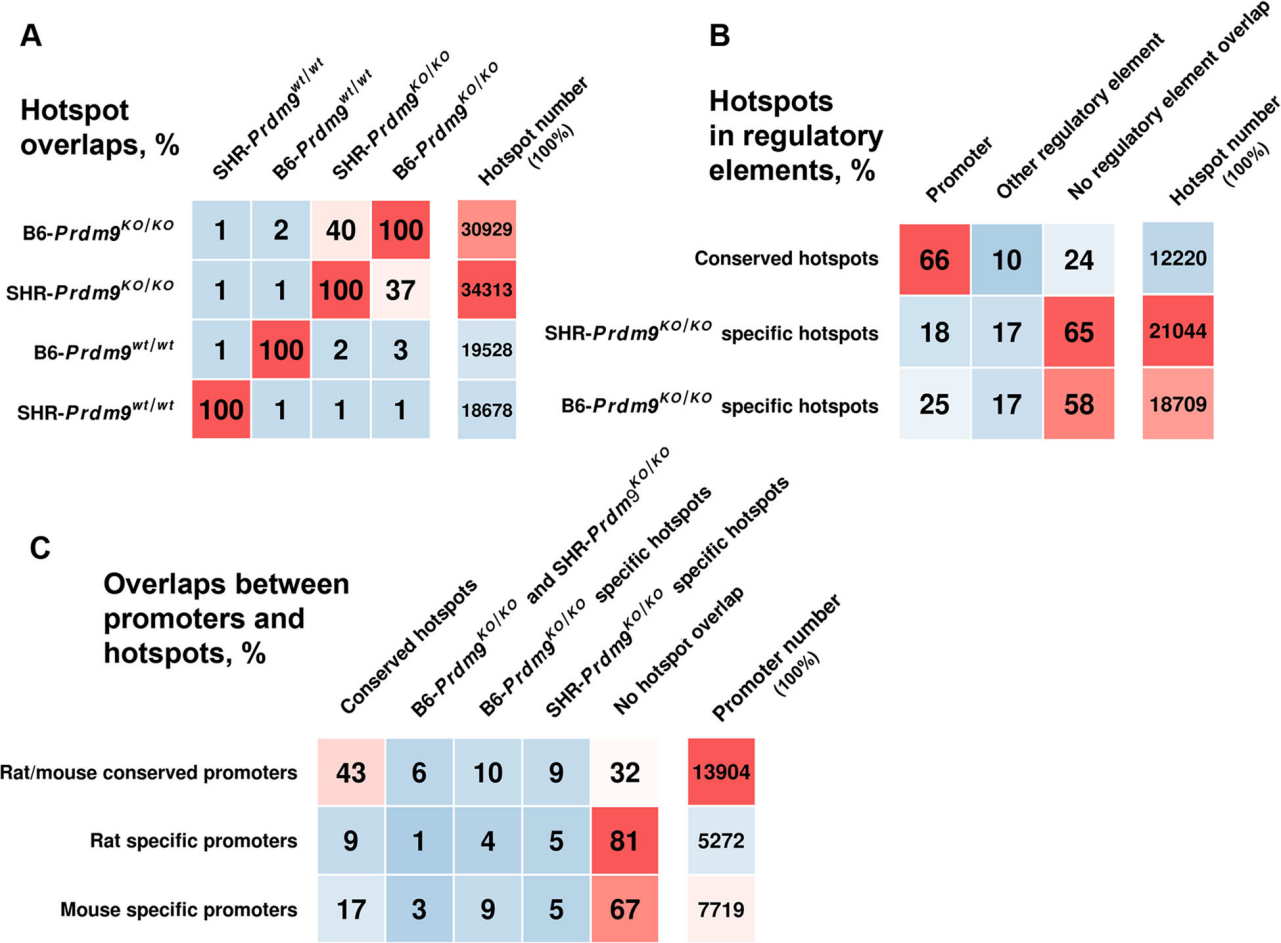
Overlaps of recombination initiation hotspots from *Prdm9*-deficient rodents with **a** wild-type hotspots and **b** regulatory elements. **c** Overlaps of promoters with hotspots

To learn about the evolutionary and functional properties of DSB hotspots (HSs), we analyzed the conservation of rat (SHR) and mouse (B6) HSs by mapping the rat HSs to the mouse genome assembly (see the “Methods” section). As expected from the analysis done in the mouse [14, 15], overlaps between wild-type HSs and *Prdm9*^*KO/KO*^ HSs were only 1 to 2% between the species (Fig. 11a). However, 40% of B6-*Prdm9*^*KO/KO*^ HSs corresponded to 37% of SHR-*Prdm9*^*KO/KO*^ HSs (Fig. 11a). Therefore, the recombination initiation maps were similar in the two PRDM9-deficient rodent species. This is analogous to different species in finches and budding yeast that naturally lack *Prdm9* [17, 18].

In general, 36% (11,888 of 33,660) of rat and 41% (12,716 of 30,929) of mouse *Prdm9*-independent HSs localized to evidence-based promoters (defined by Ensembl, see the “Methods” section), in contrast to wild-type HSs from SHR rats (343 of 19,967 or 2%) or B6 mice (900 of 19,528 or 3%). Our analysis revealed that 66% of conserved *Prdm9*-independent HSs overlapped promoters, in contrast to 25% of mouse-specific and 18% of rat-specific *Prdm9*-independent HSs (Fig. 11b). When the set of promoters was narrowed to promoters of coding genes, 27% (8229 of 30,929) of mouse HSs and 23% (7687 of 33,360) of rat HSs fell within their coordinates.

The general analysis of promoters of coding genes revealed that 65% (14,237 of 21,956) of mouse promoters overlapped with 73% (14,447 of 19,719) of rat promoters (see the “Methods” section). Sixty-eight percent of the overlapping promoters contained HSs in either one or both rodent species, and 43% of conserved promoters overlapped conserved hotspots (Fig. 11c).

## Discussion

We show that PRDM9 directs most sites of meiotic DSBs in the male rat, as it is in the mouse [39, 40] and man [5]. The relocation of rat DSBs to default (*Prdm9*-independent) H3K4me3 sites upon deletion of *Prdm9* occurs in the B6 laboratory mouse [15], where *Prdm9* deficiency causes complete sterility [12]. The partial fertility of the *Prdm9*-deficient SHR rats therefore might be due to faster and/or more efficient DSB repair of the default H3K4me3 sites in *Prdm9*-deficient rats compared to mice. One mechanism of generating mature germ cells could be bypassing the pachytene checkpoints with unrepaired DSBs. However, this would lead to aneuploid germ cells and defective offspring, while the *Prdm9*-deficient SHR animals produced fertile pups of normal appearance. Moreover, our examination of male DSB repair suggested that many DSBs are being repaired and even generate a normal number of crossovers. A comparison of *Prdm9*-deficient mice with various genetic backgrounds that differ in fertility revealed a significantly higher crossover rate (COR) in sperm-producing versus azoospermic males [14]. All 19 mouse autosomes are acrocentric; this implies that mouse COR per autosome and per autosomal arm are identical. Here we show that both wild-type and *Prdm9*-deficient SHR males display on average 27.4 autosomal crossover nodules per spermatocyte. Only eight of 20 rat autosomes are acrocentric, indicating that if one obligatory crossover per autosomal arm is required to ensure proper segregation, the COR should be at least 32 nodules per cell. We deduce that one crossover per rat autosome is sufficient (see also [41]) and that we can directly compare the autosomal rates in the mouse and rat. The mean SHR rate of 1.37 nodules per autosome (27.4/20) is more similar to the COR of sperm-producing *Prdm9*-deficient mouse males (ranging from 1.38 to 1.56) than to the COR of azoospemic *Prdm9*-deficient males (1.19 to 1.28), suggesting that COR could be associated with the fertility parameters of the *Prdm9*-deficient rat males. However, the SHR mutant carries a much higher percentage of wild-type sperm than PWD mutants (40 versus 1.2%) despite lower mean COR (1.37 versus 1.54), indicating that additional factor(s) besides COR play a role.

Unlike *Prdm9*-deficient B6 mice, where both sexes display nearly complete arrest in prophase I, PWD mutant males show only partial arrest [14], and CAST/EiJ mutant males but not females display complete arrest [26]. In contrast, both sexes of *Prdm9*-deficient SHR rat displayed age-dependent fertility and meiotic prophase I slowdown. Meiosis takes 19 days in the rat and 14 days in the mouse adult males (prophase I takes 18.5 and 13 days, respectively) [42]. The duration of female meiosis is 8 to 11 days in the rat [43] and 7 days in the mouse [44, 45]. Meiosis takes shorter time in the mouse compared to the dog, which harbors a non-functional PRDM9-encoding gene [22]: 17 days in dog males [46] and 33 to 54 days in dog females [47]. Longer meiotic prophase I gives the cell more time to synapse homologous chromosomes and repair programmed DNA breaks in order to pass the meiotic checkpoints. We therefore propose that one of the factors affecting the differences in fertility of *Prdm9*-deficient animals could be the duration of meiosis.

The SHR strain is an excellent rat model of human spontaneous hypertension [48] and might also be a better model of meiotic DSB initiation than multiple laboratory mouse strains [14], because some SHR females lacking functional *PRDM9* can produce offspring, as can at least one human female [23]. Moreover, these mutant rat females display phenotypes resembling the human syndrome Premature Ovarian Failure.

DSB targeting via mouse PRDM9 has been shown to correlate with the histone H3 methylation profiles [26, 49, 50]. We show that H3K4me3 peaks correlate with PRDM9-dependent hotspots in rat strains (Fig. 10). We found no difference between phenotypes of rats carrying truncating versus internal PR/SET-domain (catalytic) deletions, suggesting that this domain and thereby the H3K4 and/or H3K36 trimethylation are essential for PRDM9 function as in the mouse [51]. However, the mRNA with the internal deletion was produced from the same locus as mRNA encompassing truncating deletion and it has not been possible to determine the ratio of these mRNAs. Reduced protein expression of the product expected from the mRNA with internal SET/PR domain deletion was found. Therefore, we are uncertain whether or not the reason this mutation behaved as a null is because loss of 20 codons of the PR/SET domain was sufficient, or because too little PRDM9 was actually expressed. In any case, all four deletions are compatible with offspring production.

Mouse *Prdm9* has been identified as hybrid sterility gene [6–8, 52] via interaction with the *Hstx2* locus affecting COR [53–55] and via differential erosion of recombination sites [9, 39, 56–58]. Our *Prdm9*-deficient *Rattus norvegicus* could be now used to test the generality of the role of this epigenetic factor in rodent hybrid sterility.

The mouse pseudoautosomal region (PAR) encompasses PRDM9-independent DSB hotspots, while the recombination in human PARs is determined by PRDM9 [5, 15]. The rat PAR is not annotated in the reference genome assembly. Thus, it is not possible to assess the DSB activity in or adjacent to the rat PAR using our short-read sequencing approaches. The rat PAR may not be structurally analogous to the mouse PAR [59]. Only three DSB hotspots were shared among all three wild-type strains and the *Prdm9*^*KO/KO*^ rats. However, since we cannot study PRDM9-independent hotspots at or near the PAR in rats, we may be missing the primary locus at which such hotspots occur.

The two rat PRDM9 wild-type alleles characterized here are the alleles of the parental strains of BXH/HXB recombinant inbred strains, BN-Lx/Cub and SHR [48]. The fecundity of the SHR *Prdm9*-deficient rat offers the possibility to extend the current panel by strains that use new recombination sites (including promoters). These new strains could help to refine the resolution of rat mapping projects.

In addition to the anticipated apoptosis of meiotic pachytene spermatocytes, we also detected increased incidence of defective and apoptotic metaphase I/II cells and spermatids. Our result thus shows that PRDM9 is important for spermiogenesis. This increase of abnormal spermatids in rat *Prdm9* mutants could be interpreted either as an indirect consequence of meiotic defects (like in [60]) or a direct outcome of a *Prdm9* postmeiotic function. The partial metaphase and postmeiotic arrests are helpful to decipher the differences between the percentage of normal pachytene cells and the resulting percentage of normal sperm count in PWD mice and SHR rat. Metaphase arrest was previously found in several types of intersubspecific mouse hybrids (e.g., [61]); our results suggest that it may be affected by the loss of function of *Prdm9*. Age-dependent fertility has been detected in (PWK × B6)F1 mouse hybrids [6, 62]. Their delayed meiosis was rescued by manipulating the copy number of *Prdm9* [6]. We found delayed meiotic progression in our non-hybrid *Prdm9*-deficient rat strain (this report) and prepubertal PWD males [14], and thus we can interpret the delayed hybrid fertility as the result of incompatibility of *Prdm9* alleles rather than failed or dominant intergenic interaction. The delayed fertility of (PWK × B6)F1 was accompanied by a slowdown of meiosis; delayed repair of meiotic DSBs has been also been found in (PWD × B6)F1 hybrids [54]. The postmeiotic function of PRDM9 along with the reduced counts of sperm and ovarian follicles helps to explain our inability to acquire offspring from intercrosses of male and female rats both lacking PRDM9.

## Conclusions

The postmeiotic function of *Prdm9* may indicate that an additional mechanism involving PRDM9 besides repositioning meiotic hotspots may also play a role in speciation. In agreement with this conclusion, we have recently demonstrated that mouse *Prdm9* affects not just the quantity, but also the quality of sperm and spermatids generated by intersubspecific (B6 × PWD)F1 mouse hybrids [63, 64]. Mouse males captured in the hybrid zone separating the *musculus* and *domesticus* house mouse subspecies usually carry sperm, but its quantity and quality is reduced [65, 66]. The postmeiotic function of PRDM9 thus might have a similar impact on mouse speciation as the meiotic one.

## Methods

### Animals, production of *Prdm9* deletions, and genotyping

The rats were bred at the Institute of Physiology and Institute of Molecular Genetics of the Czech Academy of Sciences (permissions nos. 66/2014 and 10/2016 issued by the ethics committees), following Directive 86/609/EEC, Appendix A of the European Community Council of Europe Convention ETS123, and the Czech Republic Act for Experimental Work with Animals (Decree No. 207/2004 Sb, and Acts Nos. 246/92 Sb and 77/2004 Sb). The SHR/OlaIpcv strain was reviewed [67]. The *Prdm9* mutants were produced using mRNAs of a heterodimeric artificial zinc-finger endonuclease programmed to target the ninth coding exon of the mouse *Prdm9* gene (Sigma-Aldrich); these mRNAs were injected into fertilized SHR ova ([68]; Table 1). The *Prdm9*^*tm1YMat*^ mutant on the mouse PWD/Ph background (PWD-*Prdm9*^*tm*^) was described [14]. Genotyping PCR primers (listed in Additional file 2: Table S5) spanning the target site were then utilized to select the rats carrying *Prdm9* deletions. These deletions have been maintained on the SHR background.

### Phenotyping

Body weight (BW) and testes weight (TW) or ovary weight (OW) were measured after dissections. Sperm count (SC) was obtained after thorough mincing of the entire right epididymidis in phosphate-buffered saline (pH = 7.4) at room temperature and the count obtained using a Burker cytometer was multiplied by two. The litter size was estimated at birth; the pups were weaned at about 30 days of age. Preparation of chromosome spreads (slides with surface-spread nuclei) from testicular cells via hypotonic treatment was as published [69]. Immunocytochemistry and histochemistry were done as described [6, 7, 54, 55]; analysis of apoptosis is detailed below. The recombination nodules were counted using CDK2 immunocytochemistry on SYCP1-staged pachytene spermatocytes after subtracting the number of telomeric CDK2 foci. Antibodies are listed in Additional file 2: Table S6. Total RNAs were utilized to quantify mRNA expression levels by real-time qRT-PCR in LightCycler [7, 8] using primers shown in Additional file 2: Table S5. Evaluations of significance were done using LRM or GLM.

### PRDM9 protein detection in nuclear fractions by Western blotting

Mouse (12 dpp) and rat (18 dpp) testes were homogenized in hypotonic buffer (10 mM HEPES, pH 8.0, 320 mM sucrose, 1 mM PMSF, 1× Complete protease inhibitor cocktail EDTA-free (#11873580001, Roche) and 1× phosphatase inhibitor cocktail (#78420, Thermo Scientific) in a glass Douncer. Six and two testes were used for the mouse, and rat extracts, respectively. Testes suspensions were centrifuged at 1000×*g* at 4 °C for 10 min. Pellets were resuspended in RIPA buffer (50 mM Tris–HCl, pH 7.5, 150 mM NaCl, 1 mM EDTA, 1% NP-40, 0.5% Na-deoxycholate, 0.1% SDS, 1× Complete protease inhibitor EDTA-free and sonicated (4 cycles of 15 s ON, 15 s OFF, high power) in a Bioruptor Next-Gen sonicator (Diagenode). Suspensions were centrifuged at 16,000×*g* at 4 °C for 10 min. Supernatants were used as nuclear fractions. Nuclear fractions (10 μg for mouse and 30 μg for rat) were separated in 4–15% TBX-acrylamide gradient gel (Bio-Rad) and blotted onto nitrocellulose membranes. The membrane was blocked for at least 1 h at room temperature (1× TBST with 0.5% milk) and incubated overnight at 4 °C with affinity-purified rabbit anti-PRDM9 (1: 200) [70] or with guinea pig anti-SYCP3 (1: 3000) [70]. Secondary antibodies were goat anti-rabbit IgG-HRP (1: 5000) (#1858415, Pierce) and goat anti-guinea pig IgG-HRP (1: 5000) (#706-035-148, Jackson Immuno Research). Blots were revealed with Super Signal West Pico Chemiluminescent Substrate (#34080, Thermo Scientific). The same membrane was used for SYCP3 detection after stripping.

### Histology

Testes and ovaries were fixed overnight in Bouin’s fixative at 4 °C, washed in PBS, dehydrated in ethanol series, and stored in 70% ethanol at 4 °C. The fixed organs were embedded in paraffin, sectioned (5 μm), and mounted on glass slides. Sections were then deparaffinized using xylene and rehydrated with ethanol series. For PAS-H staining, sections were incubated with 0.5% periodic acid (Sigma) for 10 min, washed in tap water for 3 min, and stained with Schiff’s reagent (Sigma) for 20 min. Sections were counterstained with Harris hematoxylin (Sigma) for 15 s, washed in tap water for 10 min, and dehydrated with ethanol series and xylene. Whole slides were scanned and digitized with the Zeiss Axioscan.Z1 Slide Scanner with a 20× 0.8 NA objective (Carl Zeiss). The open-source software QuPath [71] was used for analyzing digital histology images of testes sections manually. Stages I to XIV of rat seminiferous epithelium cycle were determined as described [35].

### Counting and classification of apoptotic cells

Analysis of cell types undergoing apoptosis was performed in testes of 61 to 92 days old rats (three of each *Prdm9*^*wt/wt*^, *Prdm9*^*KO/wt*^, and *Prdm9*-deficient *Prdm9*^*KO/KO*^). Neighboring sections of paraffin-embedded testes were treated either with DeadEnd Fluorometric TUNEL System (Promega) or with anti-PIWIL1 primary antibody (ab12337, Abcam) followed by Goat Anti-Rabbit conjugate secondary antibody (#1706515, Bio-Rad), visualization using ImmPACT™ DAB Substrate (SK-4105, Vector Laboratories, Inc.), and hematoxylin staining. Slides were examined at 400-times magnification. Apoptotic cells were counted in TUNEL-stained sections in 200 tubules per animal; mean values were calculated from apoptotic tubules and used for further statistical processing. In addition, detailed histological examination was performed in 18 to 28 apoptotic tubules per animal to define the stages of spermatogenesis, at which cells underwent apoptosis. For this purpose, the positions of TUNEL spots were inspected in the corresponding PIWIL1-immunostained neighboring sections. Position of the apoptotic cell in the tubule, stage of the neighboring normal cells, color of cytoplasm, color and visible structural features of the nucleus, and the nucleus size, were taken into account. Mean values of total numbers of apoptotic cells per tubule, as well as of spermatocytes and round spermatids separately, were calculated from 18 to 28 tubules per animal and used for further statistical processing. Statistical analysis was performed in R using multcomp package, applying LRM and subsequently Tukey’s test. For immunofluorescent labeling of acrosome and nucleus after TUNEL detection, slides were treated with Peanut Lectin from *Arachis hypogaea* (peanut) conjugated with Alexa Fluor 568 (PNA; Invitrogen) and Hoechst 33342 (Sigma) as described [63]. TUNEL-PNA-Hoechst slides were scanned with the Zeiss Axioscan.Z1 Slide Scanner with a 20× 0.8 NA objective (Carl Zeiss) and analyzed using QuPath software [71].

### Single-strand DNA sequencing (SSDS)

DMC1 ChIP-SSDS to assess genome-wide DSB distribution using snap-frozen adult testes and its analyses were done as described previously [72, 73]. Sequencing reads were aligned to the rat rn5 genome using a modified version of bwa (0.7.12) and ssDNA-derived fragments were identified using a bespoke ssDNA identification pipeline. DSB hotspots were called using a default hotspot calling pipeline and using only uniquely mapping ssDNA type 1 fragments with an alignment *Q*-score for both paired-end reads with values over 30.

### Rat-mouse hotspot conservation and annotation

Coordinates of B6 and B6-*Prdm9*^*KO/KO*^ hotspots (HSs) were obtained from published SSDS ChIP-seq data (GEO acc. nos. GSM1954835 and GSM1954864, [39]). For detection of HSs conserved between B6-*Prdm9*^*KO/KO*^ and SHR-*Prdm9*^*KO/KO*^, we applied previously described criteria [15], considering two HSs as taking place at the same location only when overlapping within ± 200 bp distance from the HS centers. For comparative analysis of *Prdm9-*independent rat versus mouse HS, coordinates of the ± 200 bp central HS regions were converted (remapped) from rn5 to mm10 genome assembly using UCSC LiftOver binary tool [74] with default parameters, −minMatch = 0.1, and the chain rn5ToMm10.over.chain.gz. Totally, 94% of SHR-*Prdm9*^*KO/KO*^ (34,313 of 36,687) and 87% of SHR-*Prdm9*^*wt/wt*^ (18,678 of 21,589) central 400 bp HS regions were remapped. HS containing extremely long gaps after conversion were excluded from the analysis (specifically, 717 SHR *Prdm9*^*wt/wt*^ and 791 SHR-*Prdm9*^*KO/KO*^ HS with central region above 800 bp after coordinate conversion). Overlapping rat/mouse HS (central 400 nt) coordinates were merged and considered as one conserved region. HSs revealing no overlaps within central 400 nt regions were considered non-conserved, B6-*Prdm9*^*KO/KO*^- or SHR-*Prdm9*^*KO/KO*^-specific. Coordinates of rat and mouse promoters of coding genes, taken as 2000 bp upstream and 200 bp downstream from the transcription start site, were extracted from the Ensembl Genes via biomaRt R package [75]. Coordinates of rat promoters were converted from rn6 to mm10 with a chain rn6ToMm10.over.chain.gz. The overlapping rat/mouse promoter ranges were merged and considered as one conserved promoter. Location of mouse evidence-based promoters and other functional elements was taken from the Ensembl regulatory build. With the exception of coordinate conversion, all other data operations were performed in R version 3.4.4 using Bioconductor packages. Ensembl version 94 was used for exploratory analysis of conserved and non-conserved HS sets.

## Supplementary Information

**Additional file 1: Fig. S1.** Translations of mRNAs from rat *Prdm9* mutants (red) described in Fig. 1 aligned in the SET domain with homologs from other species. G278, N320 and Y341 are amino acids essential for H3K4 trimethylation activity of PRDM9 [12, 27]. **Fig. S2.** Semiquantitative qRT-PCR of adult rat testicular RNAs detects no mutant mRNA degradation. The expression values were normalized with *Actb* primers. Primer pair “Exon 1–3” detected the KRAB-encoding exons, pair “Exon 9–10” the last SET/PR-exon plus the Zn-finger-exon. Two pairs (“20 a.a. deletion”, “Intron 8”) were specific for the new mRNAs of the mutant with the largest deletion (KO516-U, KO516-L, respectively), as we did not detect them in the wild-type testes (wt/wt) or in the testes hetero- or homozygous for other *Prdm9* deletions (KO/wt, KO/KO). **Fig. S3.** Alternative views of male (**a**) and female (**b**) rat fertility parameters measured via dissections. For legend, see Figs. 2 and 3. **Fig. S4.** Impaired spermiation efficiency in *Prdm9*-deficient SHR rat. Representative images of PAS-H-stained tubular stages VIII, IX and X of *Prdm9*-deficient and control rats. Step 19 (S19) mature spermatids aligned near the tubule lumen for spermiation in stage VIII. However, some S19 spermatids were present beyond stage VIII in *Prdm9*^*KO/KO*^, indicating failed spermiation - note the abnormal retention of S19 in mutant stages IX and X. **Fig. S5.** Spermatogenic arrests in tubular stages IV and XII of *Prdm9*-deficient PWD mouse. Testicular histology using PAS-H staining. Examples of tubular stages IV and XII of *Prdm9*-deficient and control PWD males (108 dpp). Red arrows point to degenerated spermatocytes in mutant seminiferous tubules. See Fig. 4 for abbreviations. **Fig. S6.** Meiotic synapsis and DSB repair in pachytene spermatocytes of adult *Prdm9*-deficient rats detected by immunostaining of chromosome spreads- representative images. **a**-**f**, Staining of synaptonemal complex (SYCP3), asynapsed chromosomal regions (HORMAD2), and sites under repair (γH2AX). Completely synapsed autosomes were observed in normal cells (**a-d**). Sex chromosomes synapsed within the pseudo-autosomal region (**a-c**) became asynapsed during late pachynema (**d**). Aberrant spermatocytes harbored completely (**e**) and partially (**f**) asynapsed autosome pairs. **g**-**l** Labelling of SYCP1, SYCP3, and γH2AX. In normal early pachynema, all chromosomes synapsed (**g-i**); asynapsed X and Y were present at late pachynema (**j**). Abnormal spermatocytes carrying both autosomal and X/Y asynapses (**k,l**) were also detected. **m**-**r**, Immunostaining for SYCP3, centromeric proteins (CENT), and early stages of DSB repair (RAD51/DMC1). In normal spermatocytes, a few RAD51/DMC1 foci were observed on autosomes and multiple foci on sex chromosomes from early pachynema (**m-p**). All RAD51/DMC1 signal disappeared in normal late pachynema (**q**). Abnormal cells showed many strong RAD51/DMC1-stained foci on asynapsed chromosomes (**r**). **Fig. S7.** Correlations at hotspots in different rat strains. Hotspot strength is compared among all strains. Only autosomal hotspots that were called in both strains are considered. Each dot represents one hotspot and hotspot density is shown as a gray (low) to blue (high) gradient. The Spearman correlation coefficient (R2) is indicated for each comparison. **Fig. S8.** DSB hotspots are stronger on chromosome X than on autosomes. Note that since there is only one copy of chromosome X in males, hotspot strength is scaled by a factor of two.

**Additional file 2: Table S1.** Genomic and cDNA sequences. **Table S2.** Statistics for rat PAS-H data using LRM. **Table S3.** Impaired spermiation efficiency (increased ESP retention) in *Prdm9*-deficient SHR rat. **Table S4.** Quantification of cells in tubular stages IV and XII of *Prdm9*-deficient and control PWD mice. **Table S5.** List of primers. **Table S6.** List of antibodies.

**Additional file 3.** Data underlying plots and graphs in figures.

## Data Availability

The ChIP-seq and SSDS data were submitted to the GEO database under accession number GSE163474: https://www.ncbi.nlm.nih.gov/geo/query/acc.cgi?acc=GSE163474 [76].
