# Supplementary material for: Rat PRDM9 shapes recombination landscapes, duration of meiosis, gametogenesis, and age of fertility
Source: BMC Biol. 2021 Apr 28;19:86. doi: 10.1186/s12915-021-01017-0 (PMC8082845; doi:10.1186/s12915-021-01017-0)
Supplement: Supplementary file 1 — Additional file 1: Fig. S1. Translations of mRNAs from rat Prdm9 mutants (red) described in Fig. 1 aligned in the SET domain with homologs from other species. G278, N320 and Y341 are amino acids essential for H3K4 trimethylation activity of PRDM9 [12, 27]. Fig. S2. Semiquantitative qRT-PCR of adult rat testicular RNAs detects no mutant mRNA degradation. The expression values were normalized with Actb primers. Primer pair “Exon 1–3” detected the KRAB-encoding exons, pair “Exon 9–10” the last SET/PR-exon plus the Zn-finger-exon. Two pairs (“20 a.a. deletion”, “Intron 8”) were specific for the new mRNAs of the mutant with the largest deletion (KO516-U, KO516-L, respectively), as we did not detect them in the wild-type testes (wt/wt) or in the testes hetero- or homozygous for other Prdm9 deletions (KO/wt, KO/KO). Fig. S3. Alternative views of male (a) and female (b) rat fertility parameters measured via dissections. For legend, see Figs. 2 and 3. Fig. S4. Impaired spermiation efficiency in Prdm9-deficient SHR rat. Representative images of PAS-H-stained tubular stages VIII, IX and X of Prdm9-deficient and control rats. Step 19 (S19) mature spermatids aligned near the tubule lumen for spermiation in stage VIII. However, some S19 spermatids were present beyond stage VIII in Prdm9KO/KO, indicating failed spermiation - note the abnormal retention of S19 in mutant stages IX and X. Fig. S5. Spermatogenic arrests in tubular stages IV and XII of Prdm9-deficient PWD mouse. Testicular histology using PAS-H staining. Examples of tubular stages IV and XII of Prdm9-deficient and control PWD males (108 dpp). Red arrows point to degenerated spermatocytes in mutant seminiferous tubules. See Fig. 4 for abbreviations. Fig. S6. Meiotic synapsis and DSB repair in pachytene spermatocytes of adult Prdm9-deficient rats detected by immunostaining of chromosome spreads- representative images. a-f, Staining of synaptonemal complex (SYCP3), asynapsed chromosomal regions (HORMAD2), and sites under [file 12915_2021_1017_MOESM1_ESM.pdf]

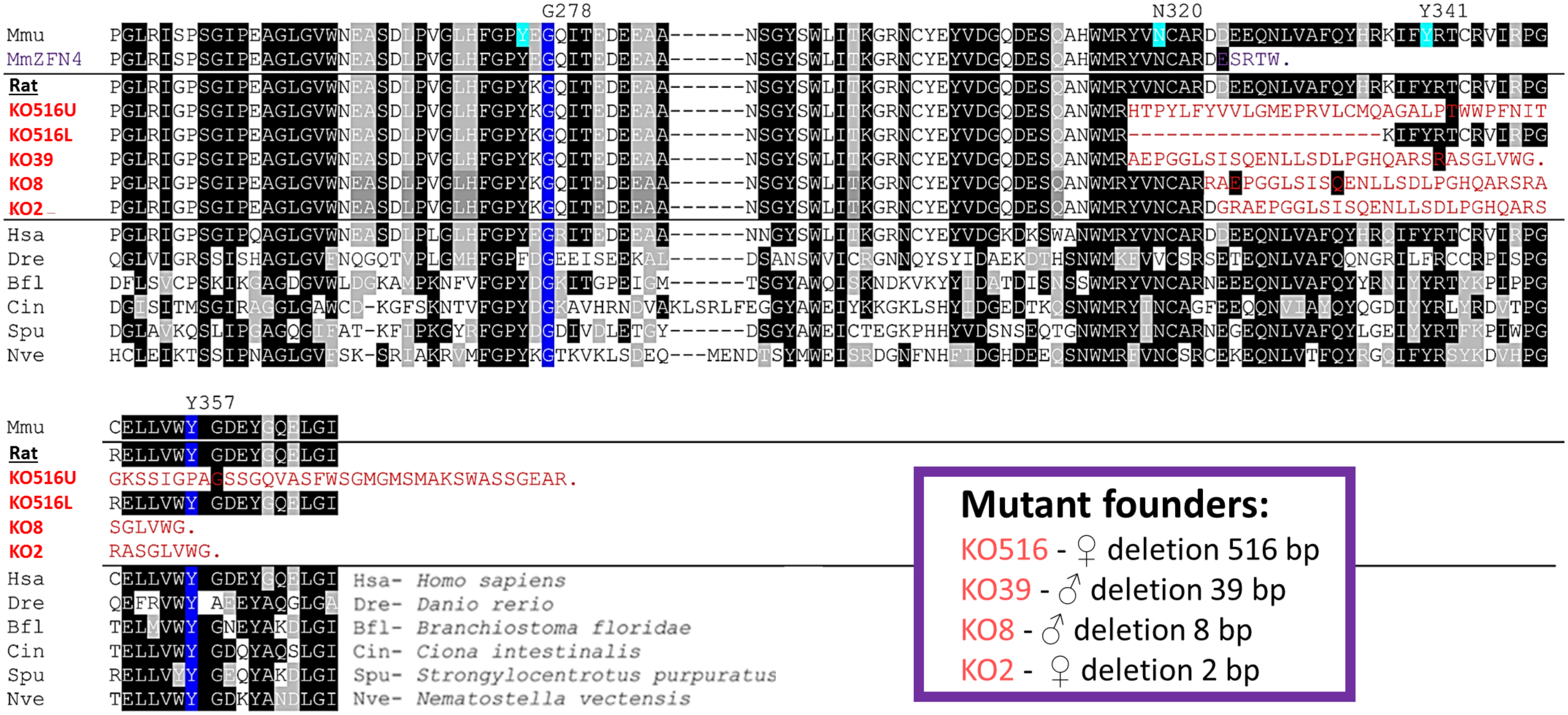

Supplemental Fig. S1

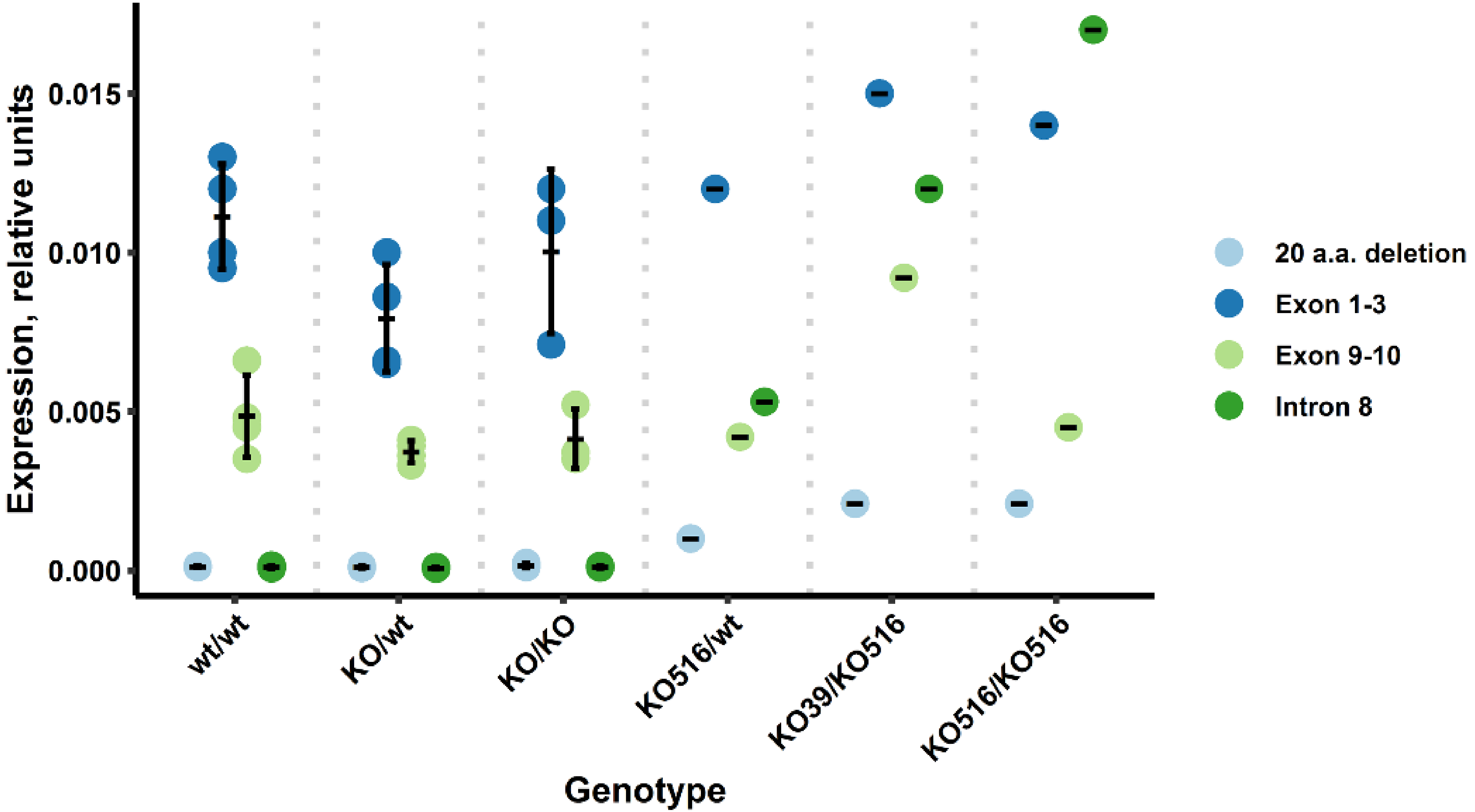

Supplemental Fig. S2

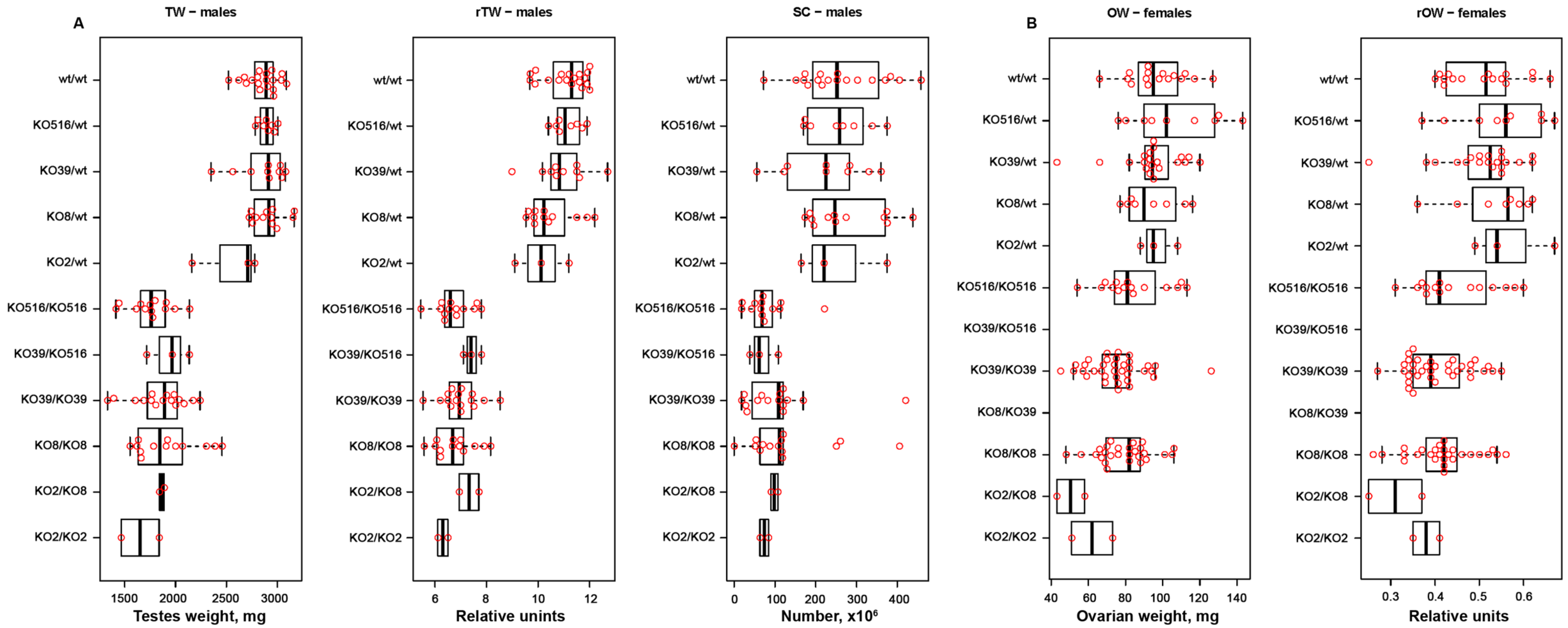

Supplemental Fig. S3

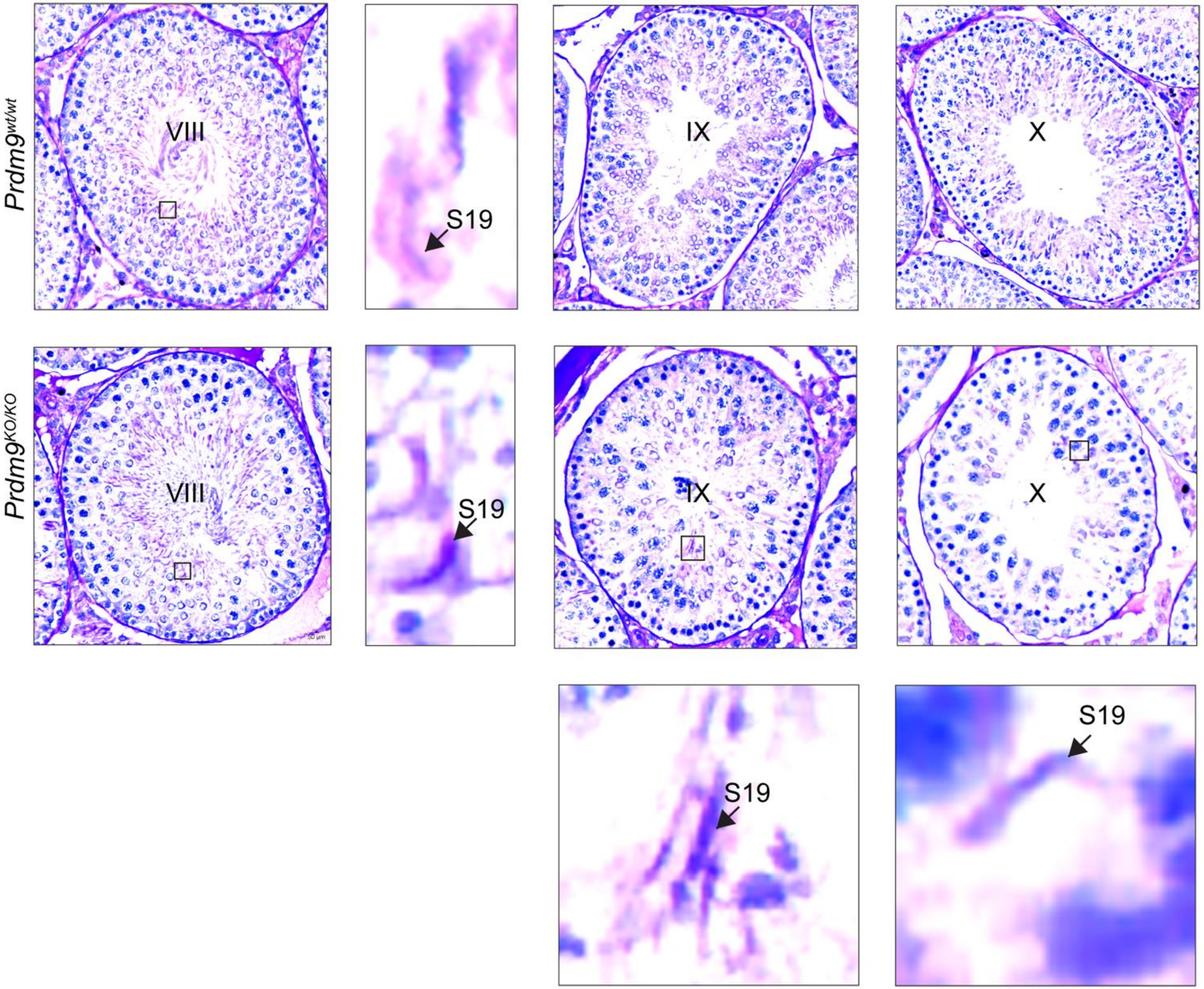

Supplemental Fig. S4

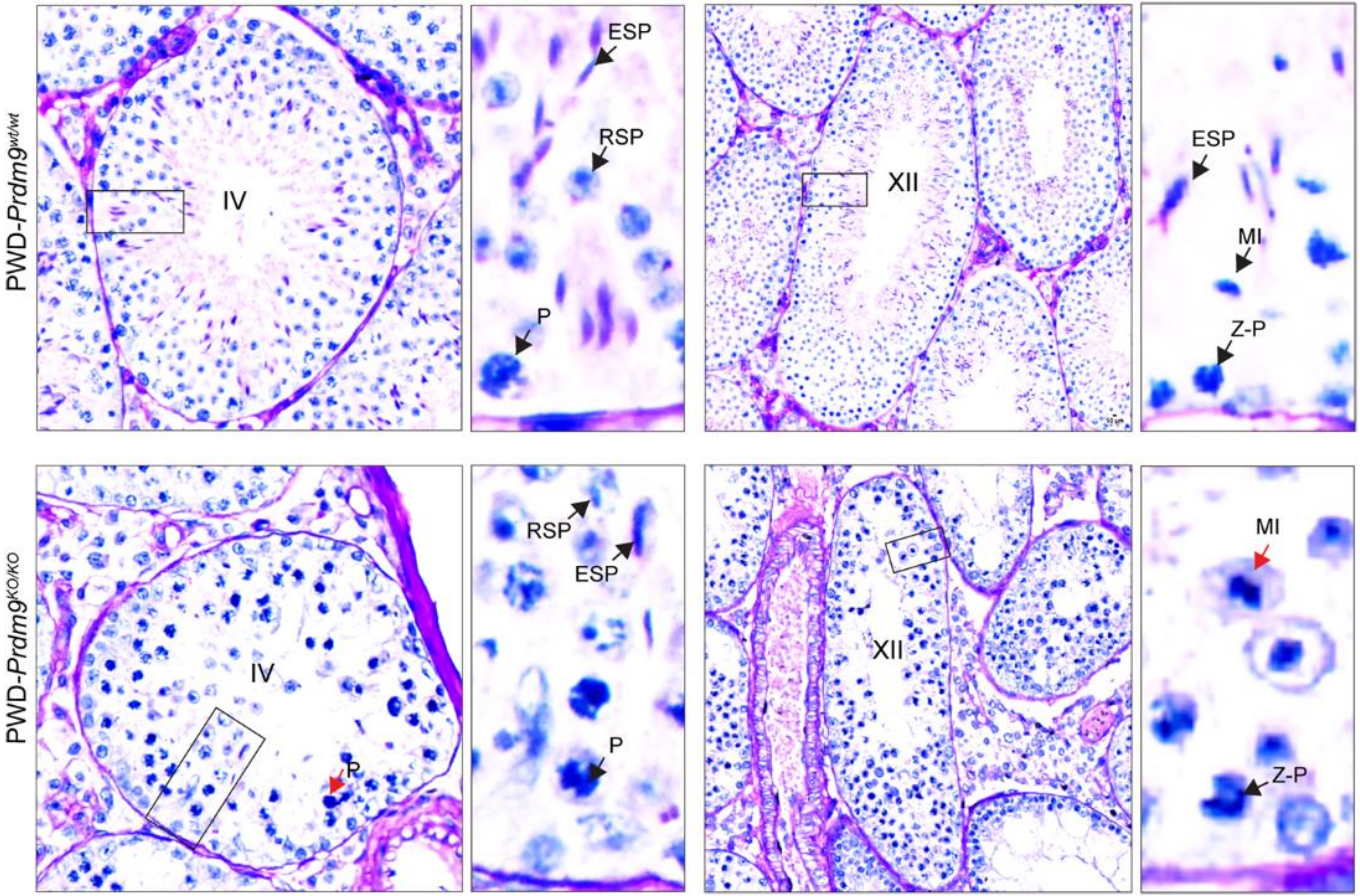

Supplemental Fig. S5

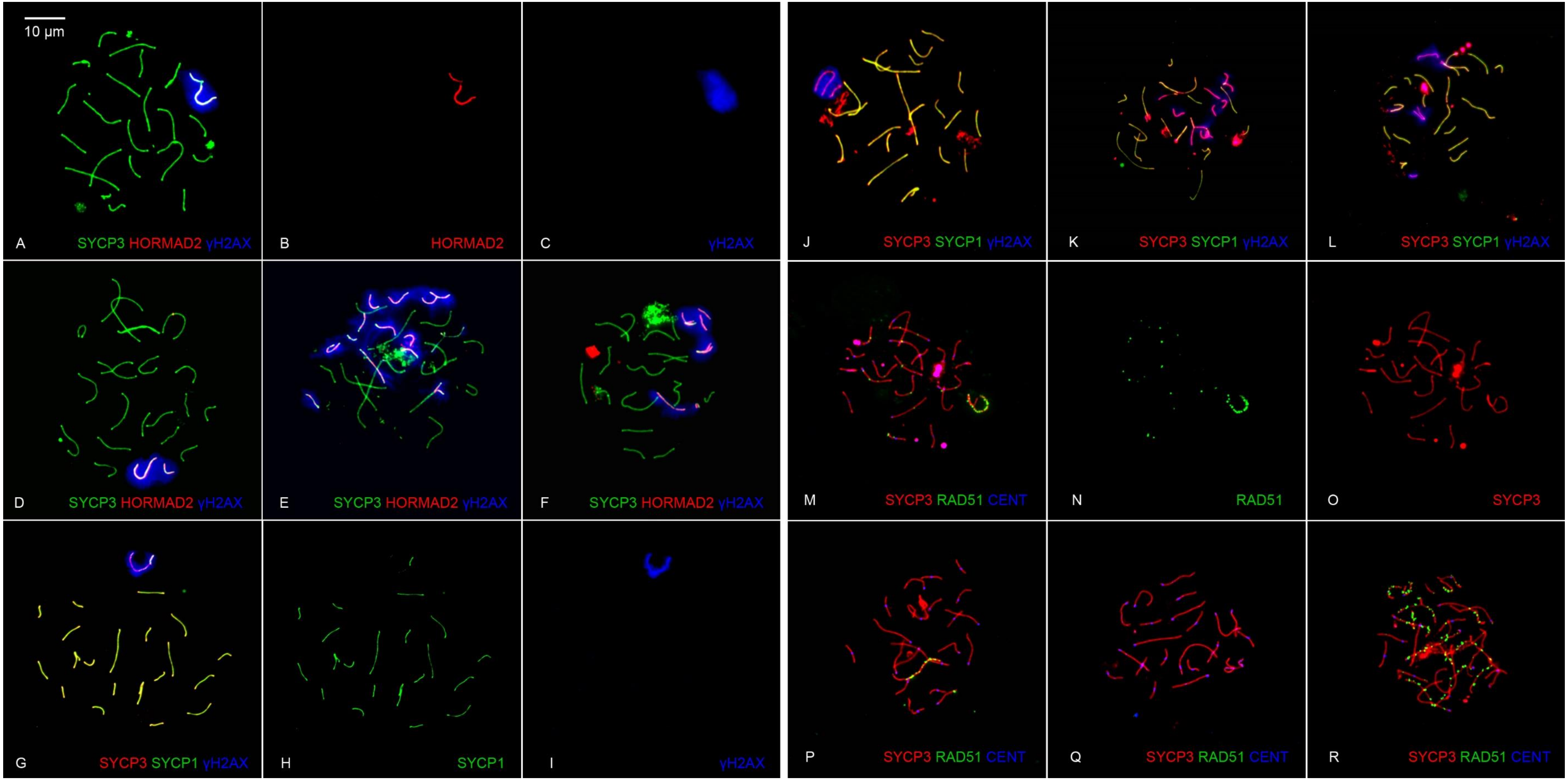

Supplemental Fig. S6

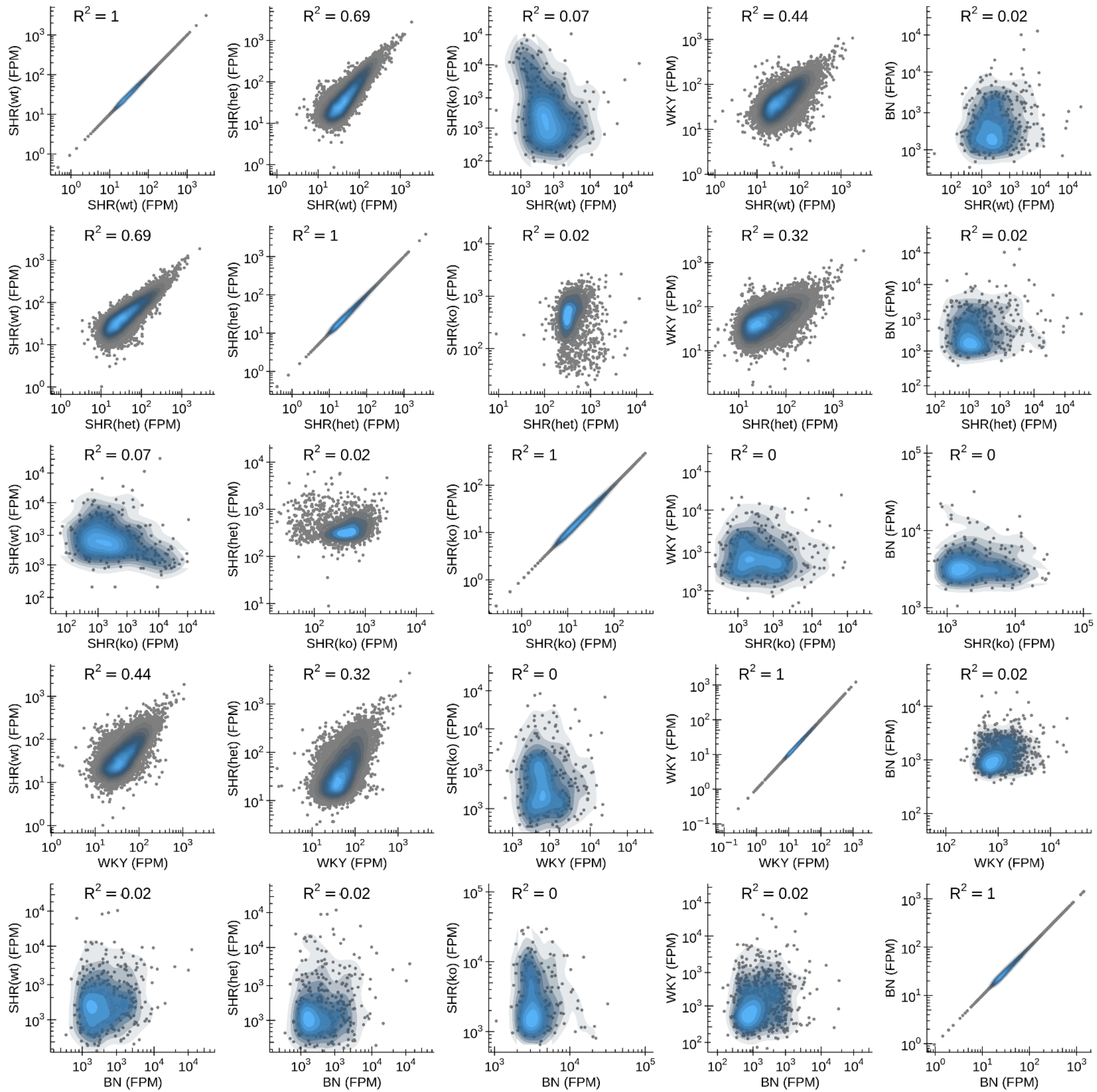

Supplemental Fig. S7

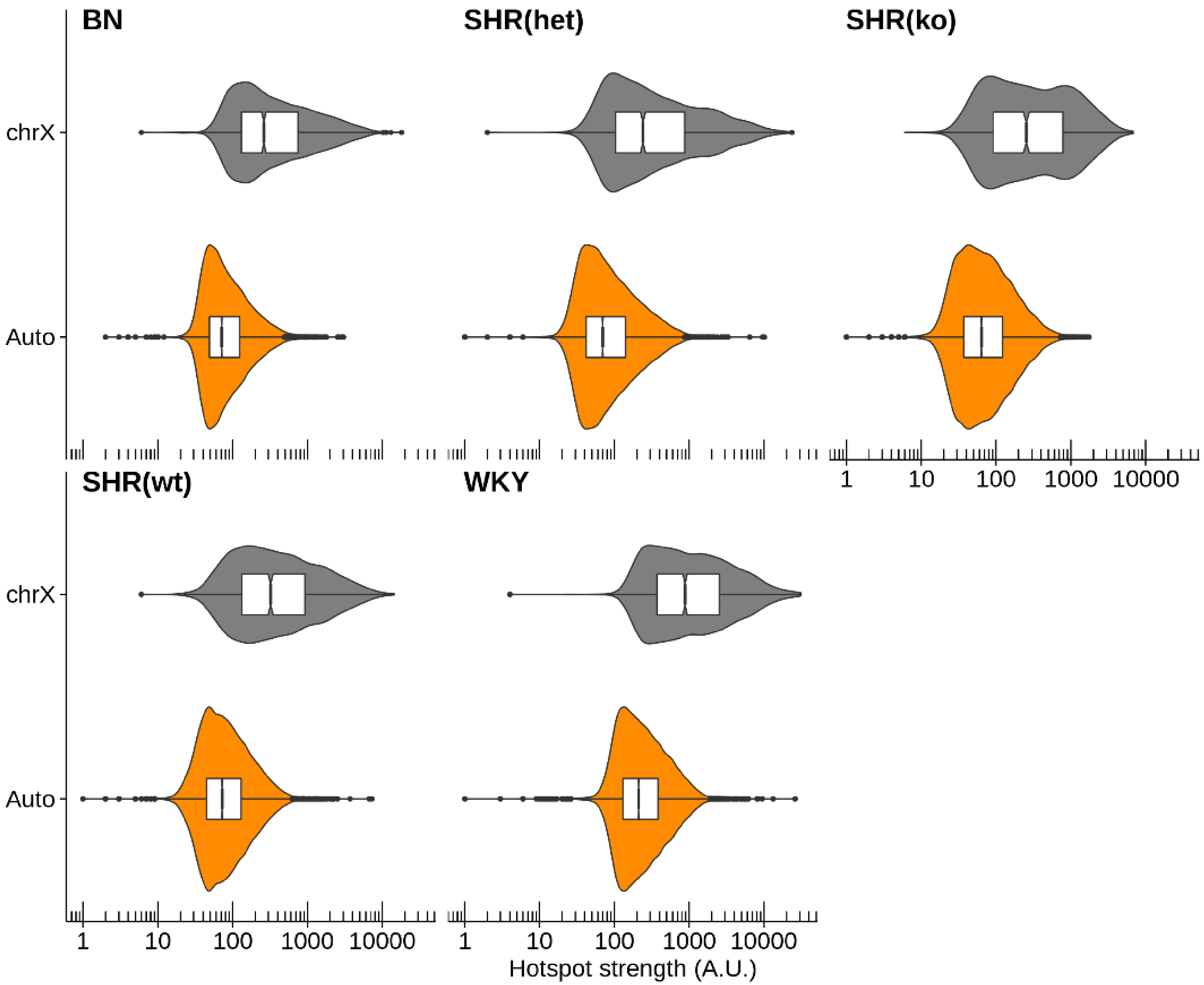

Supplemental Fig. S8
